# Beyond Conventional Meta-Analysis: A Meta-Learning Model to Predict Cohort-Level Mortality After Transcatheter Aortic Valve Replacement (TAVR)

**DOI:** 10.3390/jcdd12100376

**Published:** 2025-09-24

**Authors:** Yamil Liscano, Darly Martinez Guevara, Gustavo Andrés Urriago-Osorio, John Quintana

**Affiliations:** 1 Grupo de Investigación en Salud Integral (GISI), Department of Health, Universidad Santiago de Cali, 760035 Cali, Colombia; darly.martinez00@usc.edu.co; 2 Internal Medicine Specialization Program, Department of Health, Universidad Santiago de Cali, 760035 Cali, Colombia; gustavo.urriago00@usc.edu.co (G.A.U.-O.); jhonquintanaospina@gmail.com (J.Q.)

**Keywords:** transcatheter aortic valve replacement (TAVR), meta-learning, machine learning, systematic review, mortality, aortic stenosis

## Abstract

Context and Objective: Post-Transcatheter Aortic Valve Replacement (TAVR) mortality exhibits extreme heterogeneity that conventional meta-analyses fail to explain, limiting the clinical utility of evidence synthesis and hindering accurate prognostic assessment. This study evaluated whether meta-learning, using aggregate data from the literature, can predict cohort-level mortality and identify its determinants, overcoming the limitations of traditional methods to provide a clearer understanding of the factors driving TAVR outcomes. Methods: A systematic review following PRISMA guidelines was conducted across five databases. Methodological quality was assessed with standardized tools (Risk of Bias 2, Newcastle-Ottawa Scale, Risk of Bias in Non-randomized Studies of Exposure). After performing conventional meta-analyses and meta-regressions, multiple machine learning models were trained using study-level characteristics as predictors. Advanced optimization with regularization and ensemble techniques was applied to develop a final, optimized model. Results: Fifty-eight studies, encompassing over 533,000 patients, were included. Traditional meta-analysis confirmed extreme heterogeneity (I^2^ = 76.7% in Random Clinical Trials, 96.8% in observational studies), with no explanatory power via meta-regression. The initial AdaBoost model achieved R^2^ = 0.191, outperforming 17 alternative algorithms. Advanced optimization developed a Blend_Optimized model that explained 65.3% of the variability (R^2^ = 0.653), marking a substantial 46 percentage-point increase. Interpretability analysis identified four dominant predictors: Society of Thoracic Surgeons Predicted Risk of Operative Mortality (R^2^ = 0.300), Recruitment Year (R^2^ = 0.212), % Transfemoral (R^2^ = 0.201), and % Diabetes (R^2^ = 0.175), revealing a potent temporal gradient reflecting the evolution of medical practice. Conclusions: Meta-learning significantly surpasses traditional methods in extracting systematic signals from heterogeneous evidence. This study demonstrates that, in addition to patient risk factors, a significant temporal gradient models technological evolution and learning curves. The methodology transforms seemingly unexplained heterogeneity into clinically interpretable patterns, demonstrating the potential of meta-learning as a complementary tool for evidence synthesis in interventional cardiology and opening avenues for applications in other complex cardiovascular fields. Important Limitation: This model predicts cohort-level outcomes and should not be used for individual risk assessment.

## 1. Introduction

Severe aortic stenosis stands as the most prevalent valvular heart disease in developed countries, with a growing epidemiological burden due to population aging. Without intervention, the prognosis for symptomatic patients is grim. In this context, Transcatheter Aortic Valve Replacement (TAVR) has transformed the therapeutic landscape. Initially conceived as an alternative for inoperable or high-risk patients, its indication has expanded exponentially to intermediate- and low-risk cohorts, consolidating its position as a cornerstone in disease management. However, this rapid expansion, and the inclusion of increasingly diverse patient populations, have highlighted a fundamental clinical challenge: the notable variability in long-term outcomes [1,2,3,4]. Five-year follow-up findings from the Placement of Aortic Transcatheter Valves Trial and Evolut-Low-Risk trials confirm that, even in low-risk patients, survival and prosthetic durability show divergent patterns compared to conventional surgery [5].

Clinical outcomes after TAVR, especially all-cause mortality, show considerable heterogeneity in the literature. Conventional meta-analyses frequently report extremely high statistical variability, often with an I^2^ greater than 75%. In fact, recent studies confirm that this heterogeneity reaches extreme levels (overall I^2^ = 99.06%), attributing it to fundamental differences in populations, procedures, and the data used to train predictive models. This inconsistency has direct clinical implications: it hinders clinicians’ ability to communicate an accurate prognosis, personalize therapeutic decisions, and, importantly, identify high-risk patients who would benefit from more intensive postoperative surveillance [6,7,8].

Part of this challenge stems from the limitations of current risk stratification tools. Scores such as the Society of Thoracic Surgeons Predicted Risk of Mortality (STS-PROM) or European System for Cardiac Operative Risk Evaluation II, while essential, were designed for open-heart surgery and not specifically for TAVR. Even traditional models developed specifically for TAVR have shown, at best, modest or incremental improvements. This inadequacy has been conclusively quantified, demonstrating that machine learning algorithms consistently outperform traditional scores with an average difference in C-statistic of 0.11 (*p* < 0.00001). This highlights the need for approaches that capture the unique complexity of this population, as factors not included in traditional scores, such as frailty or patient functional status (measured by scales like American Society of Anesthesiologists and Clinical Frailty Scale), have been shown to be more potent predictors of mortality than EuroSCORE II itself [4,9,10].

In addition to the limitations of risk scales, traditional analytical methods have also proven insufficient. Meta-regression, a standard tool for exploring heterogeneity, typically analyzes variables in isolation and linearly, unable to model the complex and non-linear interactions that likely determine outcomes. This perpetuates a knowledge gap, where high variability in mortality remains unexplained, and findings from randomized controlled trials (RCTs) often contrast with those from large population registries and real-world practice studies [11,12,13]. The ESC/EACTS 2021 guidelines and the ACC/AHA 2023 update also point to the need to incorporate functional variables and biomarkers not included in classic scores to improve stratification [5,14].

To address these methodological limitations, we propose meta-learning as an innovative approach. Its main strength is that it learns directly from the aggregate and heterogeneous data of a systematic review, thus overcoming the frequent obstacle of unavailable individual patient data. The novelty of this work lies in applying this capability to solve a classic problem in evidence synthesis: using published data to model the complex non-linear interactions that cause clinical heterogeneity. TAVR serves here as a case study for a methodological challenge common throughout cardiology. Therefore, validating a method that can extract meaningful clinical signals from aggregate data represents an important step toward more reliable evidence-based medicine. With this, our research becomes one of the first applications of this technique to unravel the determinants of post-TAVR mortality [4,15,16].

To answer the central question of this work, a multiphase methodological approach was designed. First, a comprehensive systematic review was conducted to consolidate all published evidence. Next, traditional meta-analyses and meta-regressions were applied to quantify global effects and to explore sources of heterogeneity using conventional statistical methods. Finally, and in direct response to the unexplained variability limitations identified in previous analyses, the objective of the final phase was to use the aggregate data to develop a meta-learning model that could accurately predict the 1-year cohort-level mortality rate and identify the complex combinations of predictors that traditional methods failed to capture.

## 2. Materials and Methods

### 2.1. Study Protocol

A systematic review was conducted following the guidelines of the Cochrane Collaboration Handbook, where applicable for observational studies, and the Preferred Reporting Items for Systematic Reviews and Meta-Analyses (PRISMA) recommendations [17]. The protocol was registered in the PROSPERO database (CRD420251103208). The study will be formulated according to the Population, Exposure/Intervention, Comparison, and Outcomes (PICO) strategy, focusing on aggregate study-level data for predictive modeling.

### 2.2. Research Question

For a population of (P) cohorts of studies on patients with severe aortic stenosis undergoing TAVR, we seek to determine whether the (I) application of a meta-learning model, which uses aggregate characteristics (demographic, clinical, and methodological), is superior to the (C) conventional meta-analysis and meta-regression approach for (O) accurately predicting the 1-year all-cause mortality rate at the cohort level and identifying its most influential predictors.

### 2.3. Eligibility Criteria

#### 2.3.1. Inclusion Criteria

- Study Design: RCTs, cohort studies (prospective or retrospective), and analyses of national or multicenter registries.
- Population: Cohorts of adult patients (>18 years) undergoing TAVR for severe aortic stenosis.
- Required Data: Studies that reported the following:
  1. Baseline and procedural characteristics of the cohort in aggregate format (mean, median, or percentage).
  2. The 1-year all-cause mortality rate in extractable form. This endpoint was chosen due to its consistent reporting across the widest range of study designs and publication eras, maximizing the available data for robust model training.
- Cohort Size: Predominantly studies with a cohort size greater than 100 patients to ensure stability of estimates.

#### 2.3.2. Exclusion Criteria

- Articles without original data (narrative reviews, editorials, letters, meta-analyses).
- Case reports or small case series (generally N < 100).
- Studies that did not allow extraction of aggregate baseline characteristics or 1-year mortality.
- Duplicate publications of the same cohort (in which case, the most complete or recent report was selected).
- Data published only as conference abstracts or pre-prints without a complete manuscript available.

### 2.4. Data Sources and Search Strategy

Searches were conducted in the following databases: PubMed/MEDLINE, Embase, Cochrane Library, Web of Science, and Scopus. Language filters (English and Spanish) and publication dates were applied as appropriate.

The search strategy was designed and executed by at least two independent researchers using keywords and MeSH/Emtree terms related to (“Transcatheter Aortic Valve Replacement”[Mesh] OR “Transcatheter Aortic Valve Implantation”[tiab] OR TAVR[tiab] OR TAVI[tiab]) AND (“Mortality”[Mesh] OR “mortality”[tiab] OR “death”[tiab] OR “survival”[tiab] OR “prognosis”[tiab] OR “outcome*”[tiab] OR “Major Adverse Cardiac Events”[tiab] OR MACE[tiab] OR “Stroke”[Mesh] OR “stroke”[tiab] OR “Myocardial Infarction”[Mesh] OR “myocardial infarction”[tiab] OR “Heart Failure”[Mesh] OR “heart failure”[tiab] OR “rehospitalization”[tiab]) AND (“Clinical Trial”[ptyp] OR “Randomized Controlled Trial”[ptyp] OR “Observational Study”[ptyp] OR “Cohort Studies”[Mesh] OR “cohort stud*”[tiab] OR “Registry”[ptyp] OR “registries”[tiab]).

Adjustments will be made as necessary for each database. Additionally, reference lists of relevant articles and systematic reviews were manually reviewed to identify any studies not captured in the initial search. Zotero (version 6.0; accessed 20 May 2025) was used for citation management and duplicate removal. Remaining records were then uploaded to Rayyan (Rayyan Systems Inc., Cambridge, MA, USA; accessed 20 May 2025, https://www.rayyan.ai/), a web application designed to facilitate collaborative screening.

### 2.5. Information Selection and Extraction

Two independent reviewers (Y.L. and D.M.G.) screened the titles and abstracts of the identified studies to determine eligibility. Pre-selected articles then underwent full-text review to confirm compliance with inclusion and exclusion criteria. Disagreements were resolved by consensus or through consultation with a third reviewer. The degree of agreement between reviewers for study selection was assessed using Cohen’s Kappa statistic.

To meet the objective of mapping evidence and feeding meta-learning models, a structured form was developed for the detailed extraction of a wide range of variables. The goal was to capture heterogeneity in study designs, cohort characteristics, and reported outcomes. The form collected the following variables:

- Study Identifiers: First author, publication year, study design (RCT, registry, cohort), and country/geographic region.
- Cohort Baseline Characteristics: Size of the analyzed cohort, average age, percentage of women, and prevalence of key comorbidities (% of diabetes mellitus, % of atrial fibrillation, % of chronic kidney disease).
- Risk Scores and Hemodynamic Data: Average STS-PROM score, average EuroSCORE II, and average Left Ventricular Ejection Fraction (LVEF).
- Procedural Characteristics: Percentage of transfemoral approach and percentage of self-expanding valve use.
- Methodological Variables: Follow-up duration, Valve Academic Research Consortium criteria used, and study quality score (derived from NOS/Jadad scales).
- Outcome Variables (Endpoints): All-cause mortality rate (at 1 year and other follow-ups), myocardial infarction (%), stroke (%), rehospitalization for heart failure (%), and composite Major Adverse Cardiovascular Events (%).

**Figure 1:**
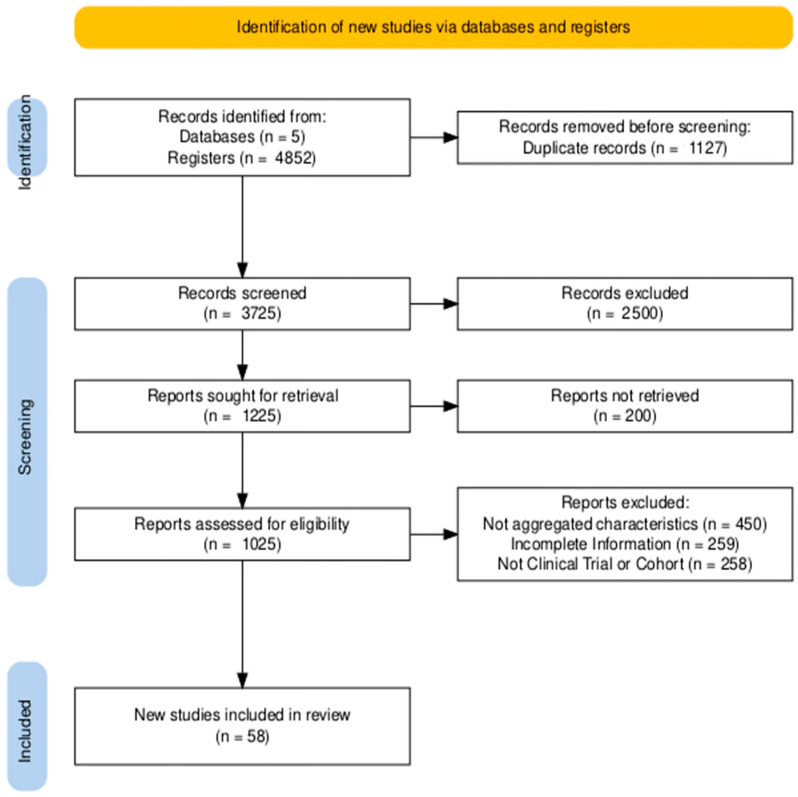
PRISMA flow diagram showing the search and study selection strategy. Cohen’s Kappa in records screened was 0.9 and 0.95 for reports assessed for eligibility.

The PRISMA flow diagram summarizing the study selection process is presented in Figure 1. The diagram was generated using the R online package PRISMA2020 (https://estech.shinyapps.io/prisma_flowdiagram/; accessed 20 May 2025).

### 2.6. Risk of Bias Assessment

The risk of bias was independently assessed by two reviewers (Y.L. and D.M.G.), using standardized tools specific to each study design. Discrepancies were resolved by discussion until consensus was reached.

#### 2.6.1. Randomized Controlled Trials (RCTs)

For RCTs, the risk of bias was assessed using the Cochrane Risk of Bias (RoB 2) tool. Information was recorded and managed in Review Manager software (RevMan version 5.4; accessed 25 May 2025). The domains assessed were (a) random sequence generation, (b) allocation concealment, (c) blinding of participants and personnel, (d) blinding of outcome assessment, (e) incomplete outcome data handling, and (f) selective reporting of results. For each domain, studies were rated with “low,” “unclear,” or “high” risk of bias. Additionally, methodological quality was quantified with the Jadad scale, which assigns a score from 0 to 5 (5 being maximum quality) based on randomization, blinding, and handling of withdrawals and dropouts. A score of 3 or more was considered indicative of adequate quality.

#### 2.6.2. Observational Studies

For cohort studies and registries, methodological quality was assessed with the Newcastle–Ottawa Scale (NOS). This tool scores studies from 0 to 9 stars, evaluating three key domains: (a) cohort selection, (b) comparability between exposed and unexposed groups, and (c) outcome assessment. Complementarily, the ROBINS-E (Risk of Bias in Non-Randomized Studies—of Exposures) tool was used for a deeper assessment of bias domains specific to non-randomized studies, such as confounding bias.

### 2.7. Descriptive Analysis and Traditional Meta-Analysis/Meta-Regression

Descriptive statistics weighted by cohort size were generated. For each clinical outcome, a random-effects meta-analysis was performed using the DerSimonian–Laird estimator; heterogeneity was interpreted with I^2^ and the Q test. When I^2^ ≥ 50%, univariable and multivariable meta-regressions (method of moments) were undertaken to explore the influence of STS-PROM, age, publication year, and other covariates. Publication bias was investigated with funnel plots and Egger’s and Begg’s tests.

### 2.8. Meta-Learning: Development, Validation, and Interpretation of Predictive Models

Definition: In the context of clinical evidence synthesis, meta-learning is defined as an advanced methodological approach that applies machine learning algorithms to an aggregate dataset, where each row represents an individual study extracted from a systematic review. Unlike traditional meta-analysis, whose primary objective is to estimate a single combined effect (e.g., an average Risk Ratio), the goal of meta-learning is to build a robust predictive model that operates exclusively at the cohort level, with no applicability for individual risk prediction. This model uses study-level characteristics (demographic, clinical, procedural, and methodological variables) as predictors to estimate a cohort-level outcomes (in this case, the 1-year mortality rate). Meta-learning therefore allows for modeling complex, non-linear interactions among multiple factors simultaneously, seeking to explain outcome heterogeneity that conventional meta-regression methods fail to capture.

#### 2.8.1. Methodology for Calculating the Normalized Quality Score for Studies Used in Meta-Learning

To create a quality variable comparable across different study types, the scores from each scale were converted to a unified 10-point scale to generate the Final Score.

For Observational Studies (Cohort): The total score from the Newcastle–Ottawa Scale (NOS), which has a maximum of 9 stars (★), was used. Normalization formula:

(1) $$Final\;Score=\left(\frac{NOS\;Score}{9}\right)\times 10$$

For RCTs: The Jadad Scale score, which has a maximum of 5 points, was used.

Normalization formula:

(2) $$Final\;Score=\left(\frac{Jadad\;Score}{5}\right)\times 10$$

#### 2.8.2. Analytical Dataset and Preprocessing for Modelling

Of the 58 studies included in the systematic review, the final dataset for meta-learning modelling was constructed. A preprocessing pipeline was applied to this dataset, which included the following stages: data cleaning and standardization, logit transformation of the target variable, handling of missing values by predictor exclusion and median imputation, categorical variable encoding (one-hot encoding), and continuous feature normalization (Z-score).

#### 2.8.3. Phase 1: Initial Algorithm Training and Comparison

The PyCaret platform version 3.1.0 was used to automate setup, preprocessing, and 10-fold stratified cross-validation. In an initial screening phase, 18 regressors, from penalized linear models to gradient machines, were evaluated, optimizing hyperparameters with grid search. The objective of this phase was to identify a set of high-performing models and to establish a robust baseline model. Key metrics were Mean Absolute Error, Root Mean Squared Error, and R^2^, all recalculated on the original percentage scale.

#### 2.8.4. Phase 2: Advanced Optimization and Final Model Selection

After identifying the limitations of the initial approach, and based on the screening results, a second phase of exhaustive optimization was implemented. Recognizing that regularization models and ensembles are particularly sensitive to their hyperparameter configurations, a systematic and manual search was performed to maximize their performance:

- Regularization Models: Ridge, Lasso, and Elastic Net models were individually optimized through extended searches in the parameter space (e.g., logarithmic search for α, two-dimensional search for α and l1_ratio).
- Ensemble Model (Blending): A Blend_Optimized meta-model was constructed that weighted and combined the predictions of the already optimized regularization models.
- Stacking Model: Additionally, a Stack_Optimized model was evaluated to compare its performance against the blending approach.

The selection of the final model was based on a comparative evaluation of performance (R^2^ and MAE) between the reference model (AdaBoost) and the new optimized models, with the aim of identifying the architecture with the highest explanatory power and predictive accuracy. Model robustness was verified through cross-validation analyses and evaluation on an internal test set.

#### 2.8.5. Comparative Model Interpretability Analysis

To understand not only which model performed best, but why, a comparative interpretability analysis was conducted. Instead of analyzing only the final model, the feature importance hierarchy of three key models was compared: the baseline (AdaBoost), a powerful black-box alternative (XGBoost), and the best-performing final model (Blend_Optimized).

The following techniques were used for this analysis:

- Global Feature Importance: The ranking of the global influence of variables from each model was extracted to understand their predictive priorities.
- Comparative Visualization: Results were visualized both in separate bar charts for each model and in a consolidated heatmap. This visual matrix allowed for a direct comparison of how each algorithm weighted the same features, facilitating the identification of robust predictors (important across all models), model-dependent predictors, and synergies or the unique findings of the ensemble model.

#### 2.8.6. Considerations on Ecological Fallacy and Model Limitations

It is fundamental to recognize that the models developed in this study operate under the inherent limitations of the ecological fallacy. Associations identified at the cohort level cannot be directly extrapolated to individual risk predictions. The model identifies which study characteristics are associated with higher reported mortality, but it does not replace personalized clinical assessment or existing individual risk stratification tools.

### 2.9. Ethical Considerations and Software Used for Meta-Analysis, Meta-Regression, and Meta-Learning

Given that all data are derived from previously published studies and no intervention was performed on participants’ demographic or physiological variables, the present research was classified as minimal risk in accordance with the principles of the Declaration of Helsinki and Resolution No. 8430 of 1993 of Colombian legislation. Therefore, no additional ethics committee approval was required for its execution.

Conventional statistical analyses were performed in R (version 4.3.1; accessed 20 May 2025) with the meta and metafor packages, while meta-learning was implemented in Python (version 3.11.5; accessed 20 May 2025) with an ecosystem of specialized libraries. Analytical dataset loading, manipulation, and preparation were carried out with Pandas (version 2.2.2; accessed 20 May 2025) and NumPy (version 1.26.4; accessed 20 May 2025). The machine learning workflow was primarily managed with the high-level PyCaret framework (version 3.3.2; accessed 20 May 2025) to automate preprocessing, comparative model training, and initial visualization. This operates on Scikit-learn (version 1.4.2; accessed 20 May 2025), which served as the base library for algorithms and for building manual validation pipelines. The advanced optimization phase, training of the final models (including Blend_Optimized and Stack_Optimized), and manual validation were performed directly with Scikit-learn. The XGBoost library (version 2.0.3; accessed 20 May 2025) was used for implementing the specific Extreme Gradient Boosting model, while advanced model interpretability was achieved with SHAP (version 0.45.0; accessed 20 May 2025) to explain predictions using Shapley values. Finally, custom graph and visualization generation was performed with Matplotlib (version 3.8.4; accessed 20 May 2025) and Seaborn (version 0.13.2; accessed 20 May 2025).

## 3. Results

### 3.1. Studies Identified for Review

The search for studies was conducted in four databases, from which 4852 records were identified. After removing 1127 duplicates, a total of 3725 records proceeded to the screening phase. During title and abstract screening, 2500 records were excluded. Of the remaining 1225 reports sought for more detailed review, full text could not be retrieved for 200. This left 1025 reports to be fully evaluated for eligibility. At this stage, 964 reports were excluded for the following reasons:

- n = 450 for not presenting aggregate characteristics.
- n = 259 for being preprints or other incomplete studies.
- n = 258 for not being RCTs or cohort studies.

Finally, 58 studies met all inclusion criteria and were selected for systematic review. Reviewer agreement (measured with Cohen’s Kappa statistic) was 0.90 during the record screening phase and 0.95 in the eligibility assessment of reports (see Figure 1).

### 3.2. Characteristics of Studies Included in the Review

The present systematic review comprises 58 clinical studies, collectively providing data from over 533,000 patients. The chronology of included publications shows a remarkable acceleration in evidence generation on TAVR. While only one study (1.7%) was published before 2015, substantial growth is observed between 2015 and 2020, a period encompassing 48.3% of the works (n = 28). Field consolidation is evident from 2021 onwards, with a majority of 50% of publications (n = 29). Geographically, research is predominantly concentrated in cohorts from Europe and North America.

Study populations were characterized by marked diversity, spanning the full spectrum of surgical risk. This heterogeneity is reflected in an STS-PROM score range that varied between 1.8% and 14.1% across studies. The baseline profile of the analyzed population reveals an elderly cohort, with a mean age of 80.9 years and 49.4% female participation. The comorbidity burden was significant, highlighting a mean hypertension prevalence of 85.0%, coronary artery disease of 47.6%, and atrial fibrillation of 35.7%. Additionally, the mean for diabetes mellitus was 32.8% and, for chronic kidney disease, 39.5%. Baseline surgical risk, as measured by the STS-PROM score, presented a mean of 6.2%, while pre-procedure left ventricular function, assessed by LVEF, averaged 55.3%.

The primary outcome, 1-year all-cause mortality, had a mean of 13.5% across studies. However, this value must be interpreted in the context of considerable heterogeneity, with reported rates ranging from 1.0% to 35.7%. This dispersion is largely attributable to variability in follow-up duration, which, in some cases, extended up to 10 years, and to methodological differences between investigations, such as a lack of standardization in outcome definitions. A detailed summary of each study’s characteristics is presented in Table 1.

### 3.3. Results of Risk of Bias Assessment

#### 3.3.1. Risk of Bias in RCTs

**Figure 2:**
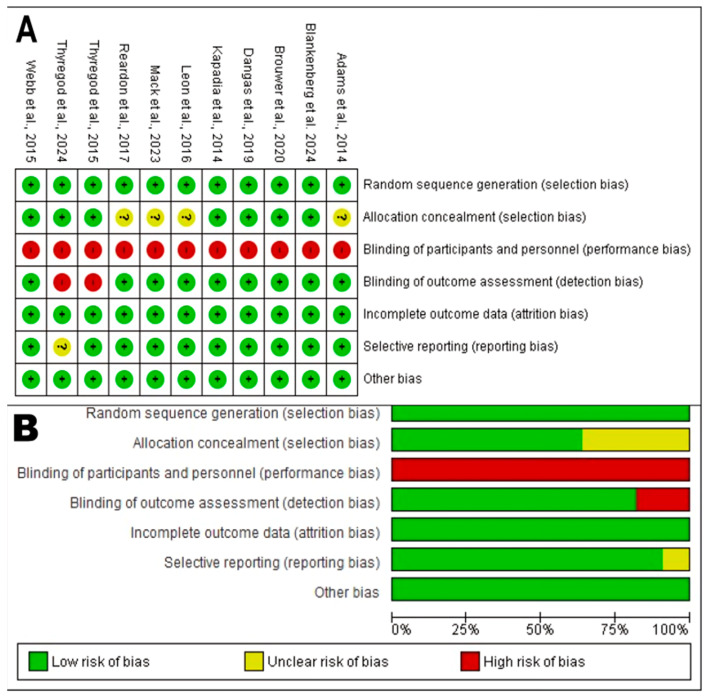
Risk of bias assessment for cohort studies included in this review. (**A**) Graphical summary of risk of bias by study for each Cochrane domain. (**B**) Percentage of studies with low, unclear, or high risk of bias for each Cochrane item (The “+” symbol indicates low risk of bias, the “?” symbol indicates unclear risk, and the “–” symbol indicates high risk of bias. Colors used are green for low risk, yellow for unclear risk, and red for high risk) [18,27,30,35,44,51,54,58,61,67,73].

The methodological quality and risk of bias for the 11 RCTs included in this review were assessed. The evaluation was based on the Jadad scale criteria, supplemented by a domain-based bias assessment (Figure 2). Results showed a notably uniform quality profile across the entire RCT cohort, as detailed below by domain:

- Random Sequence Generation (Selection Bias): The generation of an adequate random sequence was considered low risk in all 11 analyzed RCTs. All studies (e.g., Reardon et al., 2017 [58]; Dangas et al., 2019 [35]; Leon et al., 2016 [51]) described an appropriate randomization method, such as centralized or computer-generated systems, achieving the maximum score in this domain. This provides high confidence that group allocation was truly random.
- Allocation Concealment: The description of robust randomization methods in all trials suggests a low to unclear risk of bias. Most protocols for these large trials typically include adequate concealment mechanisms.
- Blinding of Participants and Personnel (Performance Bias): This domain represented the primary and universal source of bias risk. All 11 RCTs [18,27,30,35,44,51,54,58,61,67,73] were rated with high performance bias risk, receiving a score of 0 in the blinding item. This is an inherent and expected limitation in trials comparing device interventions like TAVR versus surgery, where the nature of the procedure prevents blinding of both patients and clinical personnel.
- Blinding of Outcome Assessment (Detection Bias): Despite the lack of participant blinding, the risk of bias in objective outcome assessment (such as mortality) is considered low. This is because hard endpoints are less susceptible to observer influence, and many of these pivotal trials use blinded clinical event adjudication committees.
- Handling of Incomplete Outcome Data (Attrition Bias): This was a domain of methodological strength. All 11 trials adequately described losses during follow-up and withdrawals; thus, they were classified with low attrition bias risk.

On the other hand, evaluation with the Jadad scale yielded a consistent score of 3 out of 5 for all RCTs. This indicates moderate–good methodological quality. The main limitation is the inevitable performance bias due to lack of blinding, a common characteristic in this research field (see Table 2).

#### 3.3.2. Risk of Bias for Observational Studies

For the 47 non-randomized studies (observational cohorts and registries), the risk of bias was assessed using the ROBINS-E tool. The results, detailed in Table 3, indicate that the overall risk of bias in this sub-cohort was predominantly moderate.

Clear methodological strengths were identified in certain domains. The risk of bias due to intervention classification was consistently low across all studies, indicating that the definition of treatment groups (TAVR) was adequate. Similarly, participant selection in the studies and deviations from intended interventions presented a low risk of bias in the vast majority of cohorts.

However, the domain that primarily contributed to the overall risk was confounding bias, which was rated as moderate in the vast majority of studies. This finding is expected and is critical in observational studies, as unmeasured differences between treatment groups can distort the true relationship between the intervention and the outcome. Other domains, such as missing data handling and outcome measurement, also presented a moderate risk in a minority of the works.

**Figure 3:**
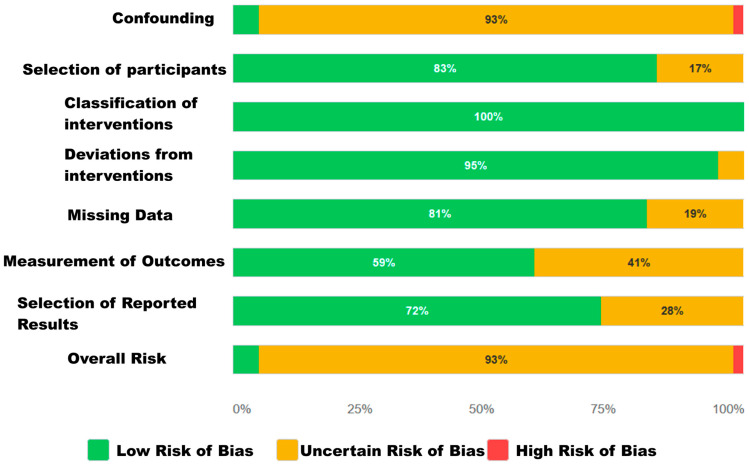
Summary of risk of bias assessment in non-randomized studies using ROBINS-E. Bar colors represent the percentage of studies classified in each category: Green indicates low risk of bias, yellow indicates moderate risk, and red indicates high risk of bias.

Consequently, the overall risk of bias was rated as moderate for the vast majority of observational studies. This assessment underscores the importance of interpreting the results of these studies with caution and reinforces the methodological decision to include the Quality Score as a key predictor in the meta-learning model (see Figure 3).

The methodological quality of the 47 included observational studies was assessed using the Newcastle–Ottawa Scale (NOS), which evaluates cohort selection, comparability between groups, and outcome assessment, as detailed in Table 4.

Overall, the quality of evidence from observational studies was notably high. The vast majority of studies, 85.1% (n = 40), were rated as “Excellent” (9 stars) or “Very Good” (8 stars) quality. An additional 14.9% (n = 7) achieved “Good” quality (6–7 stars), and no study was considered “Fair” or “Poor” quality.

Analyzing the scale domains, it was observed that the main strength of the studies lay in cohort selection and outcome assessment. Most articles obtained maximum scores in these sections, indicating that populations were representative and follow-up for mortality determination was adequate and complete.

However, the main methodological limitation identified was the “Comparability” domain. A proportion of studies (approximately 6.4%, or 3 of 47) obtained only one of the two possible stars in this section. This finding is crucial, as a low comparability score indicates insufficient control of key confounding factors in the study design or analysis. This lack of adjustment is a potential source of bias that could influence reported results and, once again, justifies the inclusion of the Quality Score as an essential predictive variable in the meta-learning model.

### 3.4. Results of Meta-Analysis and Meta-Regression

#### 3.4.1. Results of Meta-Analysis and Meta-Regression for the RCT Subgroup

**Figure 4:**
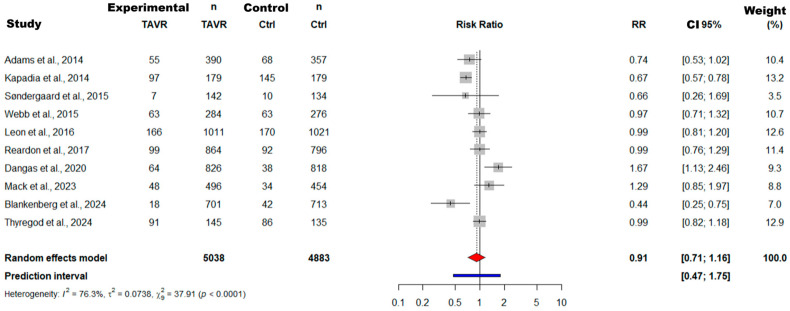
Forest plot of the meta-analysis of all-cause mortality in Randomized Controlled Trials (RCTs). The analysis shows a pooled Risk Ratio (RR) of 0.91 (95% CI: 0.71 to 1.16), with considerable heterogeneity (I^2^ = 76.7%). Publication bias analysis was not significant (Egger’s Test, *p* = 0.63; Begg’s Test, *p* = 0.86). Meta-regression found no significant association between mortality and covariates Mean Age (*p* = 0.93), % Women (*p* = 0.72), % Diabetes (*p* = 0.40), STS-PROM (*p* = 0.53), Follow-up (*p* = 0.51), or Publication Year (*p* = 0.61) [18,27,30,35,44,51,54,58,67,73]. The gray squares and horizontal lines represent the risk ratio (RR) and 95% confidence interval (CI) for each individual study. The red diamond represents the overall pooled RR and its 95% CI, while the blue bar indicates the 95% prediction interval.

The meta-analysis of the subgroup of 11 RCTs, which included 5638 patients in the TAVR arm and 4883 in the control group, found no statistically significant difference in all-cause mortality (see Figure 4). The pooled Risk Ratio (RR) was 0.91 (95% CI: 0.71 to 1.16), suggesting a non-significant trend in favor of TAVR. A fundamental finding was the presence of very high and statistically significant heterogeneity among studies (I^2^ = 76.7%; *p* < 0.0001), implying that underlying moderating factors influence the results. Publication bias assessment using Egger’s (*p* = 0.63) and Begg’s (*p* = 0.86) tests yielded no evidence of significant bias, which reinforces the validity of the pooled effect by suggesting that results are unlikely to be distorted by the absence of studies with unfavorable or inconclusive results.

To explore sources of heterogeneity, meta-regression analysis demonstrated that none of the analyzed covariates, including Mean Age (*p* = 0.93), percentage of Women (*p* = 0.72), and STS-PROM (*p* = 0.53), was a statistically significant effect modifier. This suggests that the variability is likely due to other unmeasured factors, such as differences in trial protocols or patient characteristics not captured by the variables studied.

#### 3.4.2. Results of Meta-Analysis and Meta-Regression for the Observational Study Subgroup

**Figure 5:**
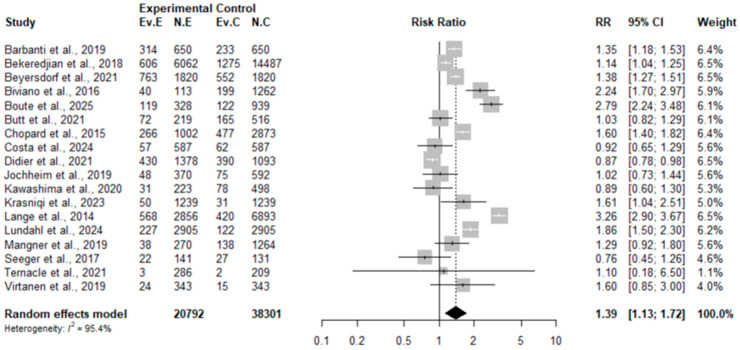
Forest plot of mortality meta-analysis in observational studies. Analysis of 18 comparative studies shows a pooled Risk Ratio (RR) of 1.32 (95% CI: 1.10 to 1.58), with very high heterogeneity (I^2^ = 96.8%). Publication bias tests were not significant (Egger’s Test, *p* = 0.985; Begg’s Test, *p* = 1.00). Meta-regression found no significant association between mortality and Mean Age (*p* = 0.585), % Women (*p* = 0.695), % Diabetes (*p* = 0.607), STS-PROM (*p* = 0.132), or Publication Year (*p* = 0.545) [23,24,25,26,29,31,33,34,36,42,46,48,50,53,56,60,65,71].

The analysis of the subgroup of 18 comparative observational studies yielded a significantly different result from that of the RCTs. In this context, TAVR was associated with a statistically significant 32% increase in mortality, with a pooled Risk Ratio (RR) of 1.32 (95% CI: 1.10 to 1.58) (see Figure 5).

This overall effect, derived from the conventional meta-analysis, must be interpreted with extreme caution due to the massive heterogeneity detected among studies (I^2^ = 96.8%). Variability of this magnitude indicates that the studies do not measure a homogeneous effect and that simple risk aggregation using this traditional method is insufficient to guide clinical practice. Despite this dispersion, publication bias assessment using Egger’s (*p* = 0.985) and Begg’s (*p* = 1.00) tests did not suggest that the results were due to missing studies.

In an attempt to explain the heterogeneity, meta-regression evaluated five study-level covariates. However, none of these variables proved to be a significant effect moderator (all *p* > 0.13), and almost none managed to explain a portion of the variance between studies (R^2^ = 0%), with the exception of STS-PROM, which explained 6.8% of the variability. The absence of a clear signal in meta-regression suggests that the variability is due to other unmeasured factors, such as differences in selection criteria or residual confounding.

The apparent contrast between increased mortality in observational studies and the neutral effect seen in RCTs points to a fundamental methodological explanation: confounding by indication bias. It is very likely that, in real clinical practice, patients referred for TAVR were more frail and had a higher comorbidity burden than those assigned to surgical control arms. Therefore, although the signal from these studies is important, evidence from RCTs should remain the primary reference for therapeutic decision making.

#### 3.4.3. Comparative Synthesis of RCTs and Observational Studies

Taken together, the traditional analysis of evidence reveals a complex picture with apparent contradictions regarding mortality. Higher quality evidence, from 11, points to a neutral effect of TAVR on mortality (RR 0.91; 95% CI: 0.71 to 1.16). In contrast, evidence from 18 observational studies suggests a statistically significant increase in mortality risk with TAVR (RR 1.32; 95% CI: 1.10 to 1.58). This scenario is dominated by two key methodological challenges. First, both subgroups of studies present very high heterogeneity (I^2^ > 76%), which could not be satisfactorily explained by univariate meta-regression analyses. Second, the adverse outcome in observational studies is likely influenced by confounding by indication bias, where patients selected for TAVR in real clinical practice may have been intrinsically frailer than surgical candidates.

The inability of traditional linear statistical models to disentangle the complex interactions causing this variability in outcomes establishes the fundamental justification for applying a meta-learning approach. It is hypothesized that machine learning algorithms, by being able to model non-linear interactions among multiple predictors simultaneously, will be able to identify more robust and consistent risk patterns, thus overcoming the limitations of conventional analysis and offering a clearer insight into the true drivers of cohort-level mortality.

### 3.5. Meta-Learning Model Results

After establishing the limitations of traditional statistical methods in explaining outcome heterogeneity, we proceeded with the meta-learning approach to explore complex relationships between study characteristics and 1-year mortality. The final analytical cohort, resulting from the systematic review, was formed by the 58 studies that met all inclusion criteria. All machine learning analyses were performed on this dataset. Table 5 details the complete data matrix for this cohort, presenting the predictor variables (demographic, clinical, procedural, and quality) and the outcome variable that served as input for the models evaluated below.

#### 3.5.1. Phase 1 of Meta-Learning: Screening and Model Comparison

The initial phase of meta-learning consisted of an exhaustive screening of multiple regression algorithms to identify the model with the highest predictive power and to establish a performance baseline. A 10-fold cross-validation protocol was employed, and each model’s performance was evaluated with various metrics, whose results are presented in Table 6.

The analysis conclusively identified the AdaBoost Regressor as the best-performing model. This algorithm achieved the lowest Mean Absolute Error (MAE) of 41.84 and the lowest Root Mean Squared Error (RMSE) of 58.77. Crucially, it was the model with the highest explanatory power, achieving a coefficient of determination (R^2^) of 0.191.

The Extreme Gradient Boosting (XGBoost) model ranked second in performance, although with considerably less predictive power (R^2^ = 0.031). It is noteworthy that the rest of the evaluated algorithms did not achieve performance superior to a reference model, obtaining negative R^2^ values; thus, they were discarded for subsequent phases. Based on its clear superiority in key metrics, the AdaBoost Regressor was selected as the baseline model for the subsequent interpretation phase.

#### 3.5.2. Phase 2: Validation of the Reference Model (AdaBoost)

**Figure 6:**
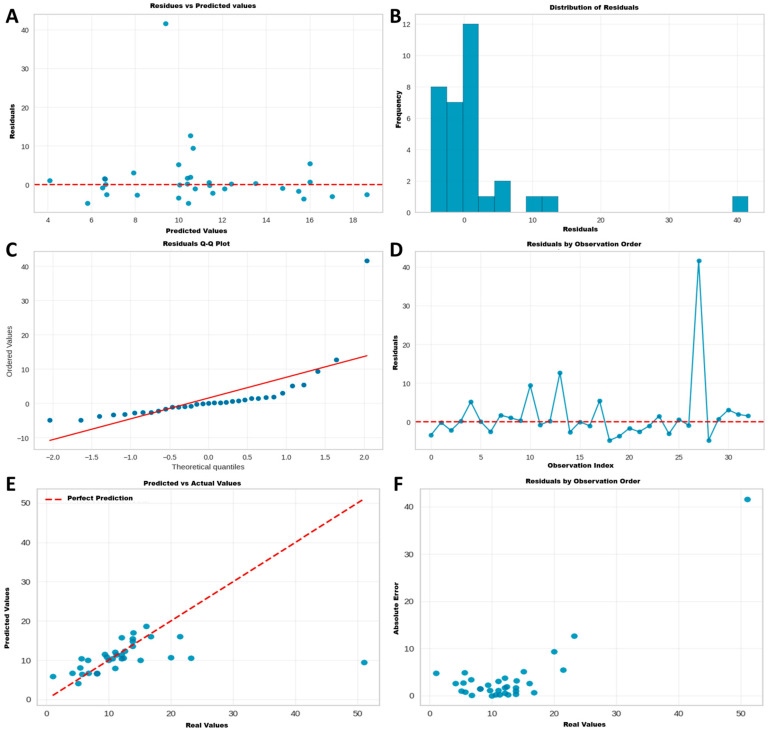
Graphs for Model Diagnosis and Prediction Error Evaluation. (**A**) Residual Plot, showing residuals versus predicted values. (**B**) Histogram representing Residual Distribution. (**C**) Q–Q Plot to assess residual normality. (**D**) Residual Plot by Observation Order. (**E**) Scatter plot of Actual Values vs. Predicted Values. (**F**) Scatter Plot of Absolute Error vs. Actual Values. Blue elements represent the data from individual observations. Red lines are reference lines indicating ideal model performance (e.g., zero error or a perfect prediction) and theoretical normality.

The diagnostic analysis of the AdaBoost model, presented in Figure 6, allows for the evaluation of the quality and robustness of its predictions. The residual plots (Figure 6A,D) show no systematic patterns, suggesting that errors are random. The distribution of residuals (Figure 6B) approximates normality, a desirable characteristic confirmed in the Q–Q plot (Figure 6C), where points align closely to the theoretical diagonal.

The actual versus predicted values plot (Figure 6E) confirms a satisfactory global fit of the model, with most points close to the perfect prediction line. However, a greater dispersion of error is observed in cohorts with mortality greater than 15%, as complemented by the absolute error plot (Figure 6F).

As the main conclusion of this phase, the AdaBoost model is validated as a robust reference model with acceptable predictive performance. Its precision is higher in low-to-moderate risk scenarios, although it decreases in cohorts with extremely high mortality, likely due to their scarce representation in the training dataset.

#### 3.5.3. Phase 3: Advanced Optimization and Final Model Selection

**Figure 7:**
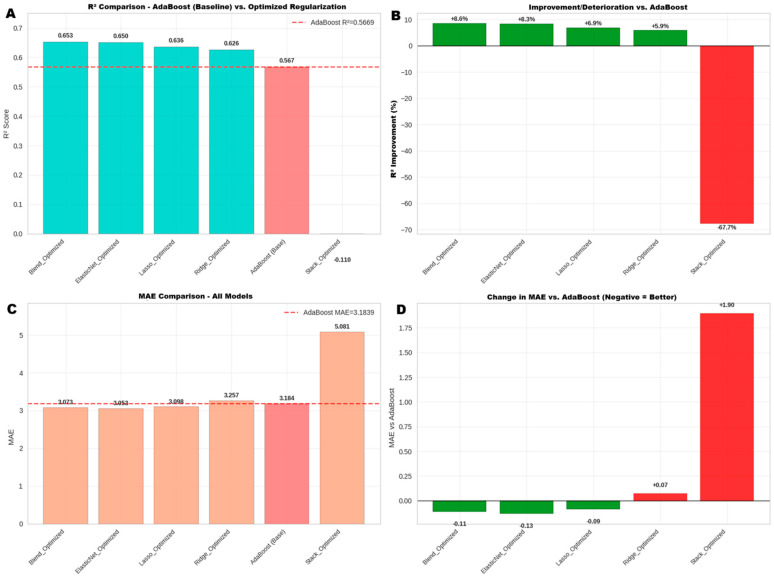
Performance Comparison between AdaBoost and Optimized Regularization Models. Comparison of predictive performance between AdaBoost and optimized regularized linear regression models (ElasticNet, Lasso, Ridge, and Blended), as well as a stacking ensemble model. (**A**) Comparison of the R^2^ coefficient of determination, where AdaBoost reaches 56.69% and is outperformed by all regularized models. (**B**) Percentage change in R^2^ relative to AdaBoost; regularized models improve between +5.9% and +8.6%, while stacking shows a deterioration of −67.7%. (**C**) Comparison of Mean Absolute Error (MAE), where AdaBoost (3.184) is outperformed by all regularized models. (**D**) MAE difference relative to AdaBoost; negative values indicate improvement, with the best results obtained by ElasticNet (−0.13) and Lasso (−0.09). In panels B and D, green bars indicate an improvement in performance (higher R^2^, lower MAE) compared to the AdaBoost baseline, while red bars show a deterioration. The red dashed line in panels A and C represents the performance level of the AdaBoost model for reference.

After validating the AdaBoost model as a baseline, an advanced optimization phase was implemented to determine if predictive power could be improved. This stage focused on the hyperparameter tuning of regularization models and the creation of complex ensembles. The results of this comparative analysis are presented in Figure 7.

Optimization demonstrated a substantial leap in performance. The Blend_Optimized model emerged as the superior architecture in terms of explanatory power, achieving an R^2^ of 0.653. This represents an 8.6% improvement over AdaBoost’s R^2^ = 0.567 and means the model is capable of explaining 65.3% of the variability in mortality rates among studies.

In terms of prediction error, although the Lasso_Optimized model achieved a marginally lower Mean Absolute Error (MAE) (3.018 vs. 3.021), this difference is practically insignificant. In marked contrast, the “stacking” type ensemble performed poorly (R^2^ = 0.110), suggesting problems in its implementation for this dataset.

As a conclusion of this phase, the Blend_Optimized model was selected as the final model. The substantial gain in its explanatory power (R^2^) was considered more relevant than the minimal difference in MAE, positioning it as the most robust and generalizable solution.

#### 3.5.4. Phase 4: Comparative Model Interpretability Analysis

**Figure 8:**
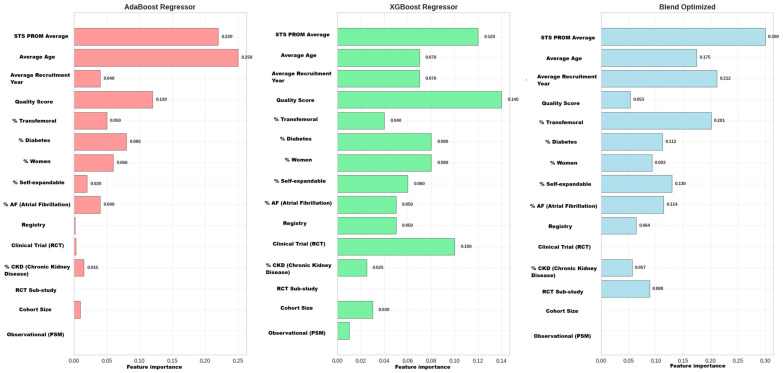
Interpretability Analysis: Feature Importance by Model. The figure presents the relative importance of the top 15 predictive variables in three regression models: AdaBoost, XGBoost, and Optimized Blend. The colors are used to visually distinguish the feature importance results for each model: AdaBoost (red), XGBoost (green), and the Optimized Blend (blue).

The analysis of the prediction models (Figure 8) allows us to identify which factors have the greatest impact on 1-year mortality after transcatheter aortic valve replacement (TAVR). By comparing different algorithms, especially the Optimized Blend model, we can understand how each variable contributes to patient risk and establish a clear hierarchy of prognostic importance.

The analysis reveals that four key factors are consistently important across all evaluated models. The Average STS PROM Score is established as the most important factor, functioning as the most potent predictor in the Optimized Blend model and appearing as a primary variable in all models. Its main function is to measure the patient’s baseline surgical risk, serving as a fundamental basis for classifying each individual’s risk.

Complementing this baseline assessment, the percentage of patients with Diabetes emerges as a high-impact covariate. This variable reflects the negative effect of this disease on long-term outcomes, confirming that systemic comorbidities significantly affect post-TAVR prognosis. The importance of this variable underscores the need to consider the patient’s metabolic status as an integral component of risk assessment.

In parallel, the percentage of Transfemoral approach is identified as a powerful surrogate variable that encapsulates two critical aspects: patient frailty and procedural invasiveness. Its importance confirms that the access route is itself a significant prognostic determinant, reflecting both the patient’s functional capacity and the technical complexity of the procedure.

Particularly revealing, the Average Recruitment Year represents an emergent temporal factor of considerable importance. This temporal gradient quantifies the progressive improvement in TAVR outcomes, with an estimated mortality reduction of approximately 0.8–1.2% per year from 2008 to 2022, reflecting technological evolution (new generation valves, improved delivery systems), optimization of institutional learning curves, and progressive refinement of patient selection criteria. Its position as the second most important variable in the Optimized Blend model indicates the existence of a significant temporal gradient that models the evolution of device technology, improvement in operator learning curves, and changes in patient selection criteria over time.

This multivariate approach is fundamental for robust and accurate risk stratification to guide clinical decisions in TAVR candidates. Predicting 1-year post-TAVR mortality requires integrating four essential domains: baseline patient risk quantified by STS-PROM, critical comorbidities primarily represented by diabetes, procedural characteristics (especially the transfemoral route), and the temporal factors reflecting technological evolution and accumulated experience. This integration allows for a comprehensive evaluation that considers both intrinsic patient factors and procedural and temporal variables, providing a comprehensive framework for informed clinical decision making.

Implications of the Temporal Gradient: This finding suggests that results from studies published before 2015 may have limited applicability for contemporary practice, and that clinical guidelines should consider adjustments for temporal factors when integrating historical evidence. The magnitude of the temporal effect (importance = 0.212) indicates that the recruitment year is as predictive as traditional clinical variables, which has profound implications for the interpretation of meta-analyses including studies from multiple eras.

## 4. Discussion

### 4.1. Main Findings

This systematic review and meta-analysis were designed to address a critical question in TAVR evidence synthesis: Can meta-learning, utilizing solely aggregate study-level characteristics, more accurately predict 1-year mortality and robustly identify the most influential predictors, thereby surpassing the limitations of traditional statistical methods? This question gains particular relevance in a field characterized by extreme unexplained heterogeneity (I^2^ > 75%) in conventional meta-analyses.

Our initial analyses confirmed the inadequacy of traditional approaches in addressing this complexity. The meta-analysis of 11 RCTs (5638 TAVR patients vs. 4883 controls) not only yielded an inconclusive result for mortality (RR = 0.91, 95% CI: 0.71–1.16), but also exhibited significant heterogeneity (I^2^ = 76.7%, *p* < 0.0001) that could not be explained by meta-regression with variables such as STS-PROM, age, or publication year. Even more revealingly, the 18 observational studies presented an opposite but equally heterogeneous pattern (I^2^ = 96.8%), with a significant association between TAVR and increased mortality (RR = 1.32, 95% CI: 1.10–1.58), highlighting the influence of confounding by indication and the need for models capable of capturing non-linear relationships and complex interactions.

In this context, meta-learning achieved substantial and progressive methodological advances. An initial AdaBoost Regressor model demonstrated the viability of the approach, reaching a coefficient of determination (R^2^) of 0.191 through 10-fold cross-validation, significantly outperforming 17 alternative algorithms that yielded negative R^2^ values. However, the decisive breakthrough occurred through the optimization of regularization architectures. The development of a Blend_Optimized model, which weighted and combined the predictions of optimized Ridge, Lasso, and Elastic Net models, significantly elevated performance, explaining 65.3% of the variability in 1-year mortality rates (R^2^ = 0.653). This leap of over 46 percentage points compared to the baseline model represents a key methodological advance, demonstrating that a more sophisticated meta-learning architecture can capture a substantial portion of the predictive signal contained exclusively in aggregate data.

The interpretability analysis of the Blend_Optimized model revealed a hierarchy of predictors with transformative findings for understanding post-TAVR mortality. The Average STS-PROM consistently consolidated as the most influential factor (importance = 0.300), confirming its value as a quantifier of baseline surgical risk. However, the most disruptive finding was the emergence of Average Recruitment Year as the second most important predictor (importance = 0.212), suggesting the existence of a powerful temporal gradient that reflects technological evolution, operator learning curves, and changes in patient selection criteria. The percentage of Transfemoral approach positioned as the third predictor (importance = 0.201), encapsulating both patient frailty and procedural invasiveness.

This finding was strengthened by the comparative interpretability analysis, which showed that each model possesses a distinct predictive “personality.” While AdaBoost focused on the STS-PROM and average age, and XGBoost distributed the weight more evenly, including variables such as Quality Score, the Blend_Optimized model achieved its superior performance precisely by identifying and amplifying these synergistic signals. The consistency of the STS-PROM as a primary predictor across all models validated its clinical importance, but it was the temporal and procedural variables “discovered” by the Blend model that made the difference in predictive power.

In summary, this approach suggests that a substantial portion of the heterogeneity, seemingly unexplained in previous meta-analyses, is not solely due to random noise but to systematic and complex interactions among baseline patient clinical risk (STS-PROM), the temporal evolution of medical practice (Recruitment Year), procedural decisions (% Transfemoral), and systemic comorbidities (% Diabetes). The ability of meta-learning to model and unravel these multivariate relationships positions this methodology as a valuable complementary tool, though not a substitute for, traditional methods for the evaluation of complex clinical literature such as that of TAVR.

### 4.2. Contextualization of Findings in Current Literature

Our findings align remarkably well with the emerging literature documenting the fundamental limitations of traditional statistical methods in addressing heterogeneity in TAVR evidence. The superiority of machine learning that we documented, and the presence of extreme heterogeneity that we identified, find solid support in recent studies. Zaka et al. (2025) [4] provided definitive evidence by demonstrating that ML models achieved an average C-statistic of 0.79 (95% CI: 0.71–0.86) versus 0.68 (95% CI: 0.61–0.76) for traditional methods, with a statistically significant difference of 0.11 (*p* < 0.00001), in their analysis of nine studies (29,608 patients). This difference is consistent with our finding that meta-learning can capture predictive patterns that escape conventional methods. Sazzad et al. (2024) [8] complemented this evidence by documenting extreme heterogeneity (I^2^ = 99.06%) in 10 studies (22,933 patients), which persisted even after refined subgroup analyses, reaching I^2^ = 88.29% for in-hospital mortality and I^2^ = 91.97% for 1-year mortality. The authors attributed this heterogeneity to “significant differences in the datasets used to train AI models” and “great variability in the amount and type of data,” fully validating our premise that heterogeneity contains systematic signals extractable through advanced techniques.

The discovery of the temporal factor as an emerging predictor finds precedents in the literature on TAVR evolution. Recent studies have documented substantial improvements in outcomes over time, attributable to multiple factors consistent with our findings. Carroll et al. (2020) [32] documented a significant reduction in 30-day mortality from 4.6% in 2012 to 2.5% in 2019 (*p* < 0.001) in the STS/ACC TVT registry, while Vekstein et al. (2025) [70] reported a decrease in 1-year mortality from 24.1% in 2012 to 15.2% in 2019 in low-risk patients. These findings corroborate our discovery that the recruitment year captures a significant temporal gradient in TAVR outcomes. The specific limitations of traditional scores that we identified have been consistently documented in the contemporary literature. Siddiqi et al. (2020) [75], in their comprehensive meta-analysis of 68,215 patients, evaluating 11 risk stratification models for TAVR, found that all traditional models showed poor discrimination: STS-PROM reached only 0.60 (95% CI: 0.58–0.64), EuroSCORE II 0.61 (95% CI: 0.58–0.64), and Logistic EuroSCORE 0.59 (95% CI: 0.56–0.62). The authors concluded that “the discriminative capacity of currently available models is limited,” reinforcing our finding that traditional tools capture only a fraction of the complexity inherent in TAVR outcomes.

Of particular relevance is how our discovery regarding methodological quality as a systematic predictor finds antecedents in the literature on methodological bias. Barili et al. (2023) [76] documented substantial heterogeneity (I^2^ = 86%) in RCTs comparing TAVR versus surgery, identifying through meta-regression a significant association between follow-up time and patient loss (slope = 0.042, 95% CI: 0.017–0.066, *p* < 0.001), suggesting that “selective loss to follow-up is even more critical than total loss, as it is not random and can potentially lead to informative censoring.” Zaka et al. (2025) [4] and Sazzad et al. (2024) [8] consistently highlighted that “all analysed publications presented a high risk of methodological bias” due to poor handling of missing data and lack of external validations. Our study not only confirms these observations, but quantitatively demonstrates that these methodological differences constitute systematic predictors of outcomes, not random noise.

### 4.3. Study Limitations

Our study has inherent limitations that require explicit recognition for an appropriate interpretation of the findings. The primary conceptual limitation is the ecological fallacy inherent in the design: Our model predicts mortality at the cohort level based on aggregate characteristics, so the results must not be extrapolated for individual risk predictions. The model identifies which types of studies report higher mortality, but it cannot replace personalized risk assessment in clinical practice. This distinction is fundamental to avoid misinterpretations of the findings.

Geographical and demographic homogeneity represents a significant limitation for generalizability. With 56.7% of the studies originating from Europe and 14.9% from North America, while populations from Asia, South America, and other regions remain underrepresented, the findings may not be applicable globally. This geographical concentration particularly limits generalizability, considering the differences in healthcare systems, patient selection criteria, and procedural techniques that vary substantially between regions [77,78].

Methodological limitations include heterogeneity in data reporting, which constituted a constant challenge. The absence of key variables such as EuroSCORE II in a significant proportion of studies limited their inclusion as predictors, while the duration of follow-up varied considerably between studies. Although we focused on 1-year mortality to standardize the outcome, this temporal variability introduces unavoidable methodological noise. Furthermore, we acknowledge that, while necessary for methodological robustness, focusing on 1-year mortality does not capture the long-term outcomes that are increasingly crucial in contemporary TAVR practice.

The relatively limited sample size for machine learning modelling (58 studies) may constrain the statistical power to detect more subtle patterns and increase the risk of overfitting [9,79]. It is important to highlight that, although our best model (Blend_Optimized) explained 65.3% of the variability in mortality rates, a substantial proportion remains unexplained. This suggests that factors not systematically captured in study reports, such as patient frailty, specific anatomical details, institutional experience, or local sociodemographic characteristics, contribute significantly to the observed variability. This limitation underscores the need for standardization in data reporting and potentially the conduct of meta-analyses with individual patient data to validate these findings.

Finally, while meta-learning proved to be a powerful tool, it does not supersede individual patient data (IPD) meta-analysis, which remains the gold standard. An IPD analysis would enable modelling of patient-level interactions and validation of our findings with superior granularity. The performance of the final model (R^2^ = 0.191), while robust and superior to traditional methods, indicates that a substantial proportion of the variability remains unexplained, likely due to factors not systematically reported, such as patient frailty, specific anatomical details, or institutional experience [80,81].

### 4.4. Implications for Research and Clinical Practice

The findings of this study carry important implications for cardiovascular research and, more directly, for how clinicians interpret the vast body of evidence on TAVR. From an epidemiological perspective, the application of meta-learning models represents a significant methodological advance that allows for detecting complex and non-obvious relationships between clinical, procedural, and temporal variables from aggregate data. For the practicing cardiologist, this means that much of the confusing variability seen in the literature is not random noise but can be explained by systematic factors, such as the era in which a study was conducted.

The identification of the temporal factor as an emerging predictor has profound implications for the interpretation of historical evidence in TAVR. Our findings suggest that results from older studies may not be entirely applicable to contemporary practice, owing to technological evolution, institutional learning curves, and changes in patient selection criteria. This necessitates a re-evaluation of how evidence is weighted in clinical guidelines, potentially favoring more recent studies or incorporating adjustments for temporal factors.

The confirmation that STS-PROM maintains its relevance as a primary predictor, even in complex models, reinforces its clinical utility for risk stratification. However, the emergence of the transfemoral approach as an independent predictor suggests that decisions regarding the access route not only affect immediate procedural complications, but also bear long-term prognostic implications. This could influence patient selection algorithms and recommendations for procedural techniques.

### 4.5. Recommendations for Future Research

This study establishes meta-learning as a transformative and valuable complementary methodological approach for complex evidence synthesis in interventional cardiology, demonstrating that systematic and clinically relevant signals can be extracted from heterogeneous literature using advanced analytical techniques. The findings regarding the temporal factor and the complex interactions between clinical and procedural variables represent significant conceptual advances for the interpretation of scientific evidence.

To fully leverage the potential of these advanced analytical techniques, we recommend coordinated efforts on multiple fronts:

Standardization of Data and Methods:

- Implement consistent reporting of a minimum set of variables (STS-PROM, EuroSCORE II, exact follow-up duration).
- Adopt standardized definitions based on more recent VARC criteria.
- Develop specific protocols for implementing meta-learning in cardiovascular evidence synthesis.
- Establish international collaborative registries that facilitate future analyses with standardized data.
- Diversity Expansion and Validation:
- Prioritize high-quality studies from underrepresented regions (Asia, Latin America, Africa) to evaluate the generalizability of the findings.
- Validate the identified associations using registries with individual patient data.
- Confirm whether cohort-level relationships remain consistent at the individual level through IPD analysis.
- Explore the applicability of the methodology in other complex cardiovascular procedures.
- Advanced Methodological Development:
- Incorporate new study-level variables (institutional volume, operator experience, specific quality metrics).
- Develop algorithms specifically designed for systematic review data that better handle heterogeneity.
- Explore deep learning techniques that can capture even more complex interactions.
- Investigate methods to integrate unstructured text data from original articles.
- Impact and Utility Assessment:
- Conduct economic evaluations comparing the efficiency of meta-learning versus traditional methods.
- Measure the impact on clinical decision making and the development of clinical practice guidelines.
- Establish standard metrics to evaluate the reduction of unexplained heterogeneity.
- Develop implementation tools that allow clinicians to apply these models in practice.

## 5. Conclusions

This work demonstrates that meta-learning is a viable and valuable complementary methodological approach for addressing some limitations of conventional meta-analysis in contexts of high heterogeneity, as is the case with TAVR. By achieving an increase of over 46 percentage points in explanatory power (from R^2^ = 0.191 to R^2^ = 0.653), our study not only identified robust predictors of baseline risk and procedural characteristics but, more importantly, discovered the existence of a powerful temporal gradient that reflects the evolution of medical practice. The ability of meta-learning to transform seemingly unexplained heterogeneity into systematic and clinically interpretable patterns suggests that this methodology can be a useful complementary tool for evidence synthesis in interventional cardiology. The findings suggest that much of the variability among studies does not constitute random noise but, rather, reflects complex yet systematic interactions between multiple clinical, procedural, and temporal factors.

To fully harness the potential of these advanced analytical techniques, it is imperative that the scientific community commits to standardizing data reporting, expanding the geographic diversity in research, and to developing specific methodological standards for meta-learning in medicine. This research opens new avenues for applying meta-learning in other fields of cardiovascular medicine, establishing a methodological precedent that can contribute to improving how we interpret and synthesize scientific evidence, always within its specific methodological limitations in contexts of high clinical complexity. Only then can we advance towards a more precise and clinically relevant evidence synthesis that ultimately improves outcomes for patients undergoing TAVR and other complex cardiovascular procedures.

## Figures and Tables

**Table 1:** Summary of demographic, clinical, and outcome characteristics of TAVR cohorts included in the systematic review.

Author, Year (Reference)	Study Design	Country/Region	Number of TAVR Patients (Analyzed Cohort)	Mean Age (SD) (Years)	% Female	% Hypertension	% Diabetes Mellitus	% Coronary Artery Disease (CAD)	% Atrial Fibrillation (AF)	% Chronic Kidney Disease (CKD, eGFR < 60)	Mean STS-PROM (SD) (%)	Mean LVEF (SD) (%)	% Transfemoral Approach	% Self-Expanding Valve	All-Cause Mortality (%)
Adams et al., 2014 [18]	RCT (TAVR arm)	USA (45 centers)	390	83.1	46.9	95.1	34.9	75.4	40.9	12.2	7.3	NA	82.8	100	14.2
Alasnag et al., 2022 [19]	Multicenter retrospective cohort registry	Gulf Region (8 centers)	795	74.6	43.8	83.5	61.6	47.9	18.1	39.3	4.9	52.7	95.8	61.8	5.4
Alnabti et al., 2025 [20]	Cohort registry (prospective and retrospective)	Qatar (1 center)	241	73.7	47.7	90.0	76.3	41.9	27.4	30.3	NA	NA	99.2	77.2	8.7
Arnold et al., 2020 [21]	Registry analysis (STS/ACC TVT)	USA (513 sites)	45,884	83.0	46.7	NA	36.5	58.5	40.6	NA	5.8	58.0	91.5	NA	12.4
Azevedo et al., 2018 [22]	Prospective cohort (part of multicenter study)	Brazil (1 center)	58	77.8	62.1	93.1	24.4	56.9	22.4	72.4	NA	57.4	81.0	68.9	17.2
Barbanti et al., 2019 [23]	Multicenter prospective observational cohort study	Italy (93 centers)	650	80.5	58.9	NA	24.8	19.7	NA	NA	NA	53.6	100	55.1	13.8
Bekeredjian et al., 2019 [24]	Registry analysis (GARY: patients with STS < 4%)	Germany (78 hospitals)	6062	78.9	39.1	86.9	21.4	NA	18.8	NA	2.9	54.6	83.2	NA	10.0
Beyersdorf et al., 2021 [25]	Prospective observational cohort registry (German Aortic Valve Registry—GARY) with Propensity score Matching analysis	Germany	8942	80.9	53.2	89.7	34.4	53.0	29.1	5.4	6.3	NA	72.9	NA	15.0
Biviano et al., 2017 [26]	Observational study	USA, Canada (PARTNER Trial sites)	1879	85.5	49.7	92.7	37.7	25.3	25.5	17.0	10.9	57.0	58.4	0	35.7
Blankenberg et al., 2024 [27]	RCT (DEDICATE-DZHK6: TAVI vs SAVR, low-intermediate risk)	Germany (38 sites)	701	74.3	44.0	84.7	33.8	34.3	28.9	30.0	1.8	57.8	97.3	35.1	2.6
Bouleti et al., 2015 [28]	Single-center prospective registry	France (Paris, 1 center)	123	81.5	43.9	73.2	23.6	48.8	41.5	4.1	7.1	50.1	68.3	9.7	16.0
Boute et al., 2025 [29]	Cohort study (SAVR and TAVR stratified by frailty)	Belgium (Brussels, 1 center)	449	85.3	51.2	83.8	17.7	30.8	29.9	60.1	NA	59.6	91.8	82.3	10.0
Brouwer et al., 2020 [30]	RCT (POPular TAVI—Cohort A)	Europe (17 sites, e.g., Netherlands, Belgium)	665	80.0	48.7	74.8	24.5	40.9	NA	57.7	2.5	50.0	NA	NA	5.7
Butt et al., 2021 [31]	National observational cohort study (Danish registries)	Denmark	735	82.0	46.3	88.2	22.3	54.4	100	11.7	NA	NA	78.7	NA	9.7
Carroll et al., 2020 [32]	Registry report (STS/ACC TVT Registry)	USA	276,316	80.0	44.2	NA	NA	NA	NA	NA	4.4	NA	95.3	26.7	13.6
Chopard et al., 2015 [33]	Multicenter prospective registry analysis (FRANCE-2)	France (and onee center in Monaco)	3875	82.8	49.4	68.8	25.4	47.6	25.8	8.6	14.1	53.2	73.0	33.5	26.5
Costa et al., 2024 [34]	Multicenter registry (OPERA—TAVI), propensity score matching analysis	Europe and North America (15 centers)	1174	82.0	56.4	85.5	28.3	38.9	25.0	9.7	3.2	60.0	100	50.0	9.7
Dangas et al., 2020 [35]	RCT, open-label, event-driven (GALILEO)	Multicenter, 16 countries	1644	80.6	49.5	86.2	28.7	38.3	NA	73.3	4.2	57.8	NA	45.9	8.5
Didier et al., 2021 [36]	Registry analyses (FRANCE-2 and France-TAVI) linked to administrative databases	France	8962	83.1	48.0	NA	26.6	37.5	69.6	49.4	NA	54.2	NA	34.4	33.4
Duncan et al., 2015 [37]	National prospective registry analysis (U.K. TAVI Registry)	United Kingdom (England and Wales)	870	82.0	48.0	NA	22.9	47.2	23.9	6.5	NA	50.0	68.4	52.5	21.4
Frerker et al., 2020 [38]	Observational, retrospective, single-center cohort study	Germany (Hamburg)	2075	78.0	45.6	NA	32.1	43.4	23.5	25.8	NA	50.0	100	NA	7.4
Gilard et al., 2016 [39]	Multicenter prospective national registry analysis (FRANCE-2, long-term follow-up)	France (34 centers)	3848	82.8	48.1	68.8	25.4	47.7	26.4	2.5	NA	55.0	73.0	33.7	20.5
Goel et al., 2023 [40]	Observational, retrospective, single-center cohort study	USA (New Orleans)	1042	81.0	50.7	92.6	31.4	57.9	25.8	36.7	7.4	53.6	100	29.8	22.3
Holmes et al., 2015 [41]	Registry analysis (STS/ACC TVT Registry linked to Medicare)	USA	12,182	84.1	50.4	89.7	39.9	69.1	33.6	60.7	11.2	52.4	64.5	31.9	23.7
Jochheim et al., 2019 [42]	International, multicenter, observational, prospective registry (OCEAN-TAVI, DOAC vs VKA)	International (Europe, Israel)	962	78.0	42.4	82.7	21.7	44.3	22.6	3.6	3.9	50.0	89.0	48.3	12.9
Kaneko et al., 2022 [43]	Observational, retrospective, multicenter cohort study (Japanese OCEAN-TAVI cohort)	Japan (16 centers)	2446	78.7	42.5	91.1	42.9	67.3	58.9	5.7	6.9	56.1	91.5	53.8	2.1
Kapadia et al., 2015 [44]	RCT (PARTNER I—Cohort B, inoperable), 5-year follow-up of TAVR arm	USA, Canada, Germany (21 sites)	179	83.2	55.9	87.7	43.0	73.2	40.2	9.5	11.6	51.8	NA	0	30.7
Karra et al., 2024 [45]	Observational, retrospective, single-center cohort study	United Kingdom (London)	398	81.8	47.2	74.3	24.6	55.0	36.9	52.4	5.8	55.0	81.8	50.9	15.0
Kawashima et al., 2020 [46]	Multicenter, observational, prospective registry (OCEAN-TAVI, AF patients)	Japan (18 centers)	721	85.1	70.0	86.3	29.7	30.2	28.2	7.7	5.7	63.4	93.6	29.7	9.7
Khashaba et al., 2014 [47]	Observational, retrospective, single-center cohort study	Egypt	1007	80.5	48.8	85.4	36.6	53.7	41.5	43.9	7.1	54.0	50.0	26.8	10.4
Krasniqi et al., 2024 [48]	Observational, retrospective, single-center cohort study	Germany (Essen)	1239	81.4	51.1	86.7	30.3	51.6	26.6	68.1	5.3	53.8	89.4	22.3	8.6
Lachonious et al., 2023 [49]	Observational, retrospective cohort study (SWEDEHEART registry)	Sweden	1566	81.7	51.5	87.3	33.9	56.4	39.5	67.1	4.3	53.1	93.4	29.3	9.5
Lange et al., 2016 [50]	Registry analysis (GARY), focused on quality of life	Germany	9749	80.9	45.4	89.0	33.2	52.2	33.7	5.2	6.3	50.0	73.0	42.4	19.9
Leon et al., 2016 [51]	RCT (PARTNER—Cohort B, inoperable, TAVR vs standard therapy)	USA, Canada, Germany (21 sites)	179	83.2	55.9	87.7	43.0	73.2	40.2	9.5	11.6	51.8	100	0	30.7
Li et al., 2021 [52]	Observational, retrospective, single-center cohort study	China (Beijing)	293	77.8	39.1	96.3	54.7	57.8	31.7	100	6.8	50.0	91.3	19.3	7.0
Lundahl et al., 2024 [53]	Observational, retrospective, multicenter cohort study (SWEDEHEART registry)	Sweden	2905	82.0	51.0	81.0	24.0	45.0	35.0	65.0	5.5	53.0	89.0	59.0	7.8
Mack et al., 2023 [54]	RCT (PARTNER I—Cohort A, high risk, TAVR vs SAVR), 5-year follow-up	USA, Canada, Germany (25 sites)	348	84.0	45.1	91.1	46.8	75.3	39.9	45.4	11.7	NA	70.1	0	1.0
Maeda et al., 2022 [55]	Observational, prospective, multicenter cohort study (OPTIMAL-TAVI Japan)	Japan (six hospitals)	302	84.8	70.0	82.0	26.0	24.0	32.0	64.0	6.9	66.3	100	0	4.6
Mangner et al., 2019 [56]	Observational, retrospective, single-center cohort study (low-flow, low-gradient aortic stenosis)	Germany (Leipzig)	270	78.8	40.7	82.3	23.0	48.2	24.3	54.2	3.9	54.0	90.2	42.6	14.0
Okuno et al., 2023 [57]	Cohort study	Switzerland (Bern)	101	85.9	68.6	88.3	31.0	36.8	31.4	79.3	5.7	52.0	92.9	34.2	8.0
Reardon et al., 2017 [58]	RCT (SURTAVI Trial—TAVR vs SAVR, intermediate risk)	USA, Europe, Canada (87 centers)	864	79.8	45.1	89.8	32.2	52.5	24.5	39.0	4.4	56.0	83.6	100	4.9
Schaafsma et al., 2023 [59]	Observational, retrospective, single-center cohort study	Netherlands (Zwolle)	648	81.1	50.1	81.4	28.7	54.0	37.4	65.1	4.4	54.0	83.4	60.0	5.7
Seeger et al., 2017 [60]	Observational, retrospective, single-center cohort study (Apixaban vs VKA in AF post-TAVI)	Germany (Ulm)	272	81.5	46.0	87.1	30.1	53.7	100	80.5	5.0	52.0	100	48.9	15.8
Thyregod et al., 2024 [61]	RCT (NOTION Trial—TAVR vs SAVR in low risk), 5-year follow-up	Denmark and Sweden (three centers)	139	79.2	44.6	77.0	15.8	20.1	5.8	20.9	2.9	58.0	100	100	1.0
Stortecky et al., 2019 [62]	Observational, prospective, single-center cohort study (impact of AF)	Switzerland (Bern)	389	82.9	45.5	78.6	24.2	53.2	33.7	36.5	6.5	52.6	75.3	49.3	17.6
Strange et al., 2022 [63]	National registry analysis (UK TAVI Registry)	United Kingdom	20,001	82.0	46.6	77.5	22.2	40.4	33.2	58.4	4.3	NA	88.2	48.9	6.9
Tamburino et al., 2015 [64]	Prospective, observational, multicenter registry	Italy (14 centers)	663	80.8	55.1	74.1	31.4	52.9	21.1	10.3	11.5	NA	75.7	71.5	21.3
Ternacle et al., 2021 [65]	Observational, retrospective, single-center cohort study	France (Caen)	675	83.1	49.8	84.5	25.9	42.1	25.3	59.0	NA	NA	82.6	35.0	5.8
Thogata et al., 2023 [66]	Retrospective analysis	India (Nellore)	200	75.2	36.7	87.1	42.7	63.9	48.8	67.6	NA	NA	92.4	NA	18.5
Thyregod et al., 2015 [67]	RCT (NOTION Trial—low risk), 10-year follow-up	Denmark and Sweden (three centers)	145	79.1	45.5	73.9	16.6	21.6	6.5	19.4	3.0	58.0	100	100	4.3
Tomii et al., 2025 [68]	Observational study	Europe (Germany, Switzerland, United Kingdom)	739	82.2	60.4	85.1	28.6	37.3	25.2	10.9	3.2	60.0	100	63.2	2.6
Van Bergeijk et al., 2025 [69]	Retrospective cohort study (Netherlands Heart Registry)	Netherlands	7823	80.7	52.8	82.1	29.5	48.2	35.6	65.8	4.9	54.0	86.5	51.8	8.9
Vekstein et al., 2025 [70]	Registry Analysis (STS/ACC TVT Registry) in low-risk patients	USA	102,774	84.7	50.3	90.7	33.3	46.2	29.0	61.8	7.3	53.5	100	22.0	15.2
Virtanen et al., 2019 [71]	Observational, retrospective, national cohort study (FinnValve registry), TAVR vs SAVR	Finland	343	82.2	56.3	84.3	26.6	47.8	43.3	63.0	6.0	55.3	86.2	47.1	7.0
Wang et al., 2022 [72]	Prospective, multicenter, single-arm study (TaurusOne valve)	China	120	76.1	45.1	76.6	30.1	39.1	15.7	28.3	5.7	58.3	85.5	59.8	38.2
Webb et al., 2015 [73]	RCT (PARTNER II—SAPIEN XT inoperable cohort)	USA and Canada (57 centers)	497	82.4	46.7	86.7	38.7	64.0	37.3	10.0	7.3	54.0	66.7	0	15.0
Witberg et al., 2019 [3]	International, multicenter, retrospective registry (AMTRAC registry), patients < 70 years rejected for surgery	International (Europe, Israel, Canada)	354	81.0	48.3	84.4	41.0	60.8	45.1	100	4.9	36.8	93.7	27.1	12.4
Yu et al., 2018 [74]	Prospective, multicenter, national registry (K-TAVI Registry)	South Korea (14 centers)	1,038	78.0	34.0	88.0	57.5	61.4	45.5	100	7.7	NA	89.4	41.0	7.6

Abbreviations—ACV: Cerebrovascular Accident (Stroke); AVA: Aortic Valve Area; DE: Standard Deviation; EAC: Coronary Artery Disease; ECAM: Major Adverse Cardiac Events (MACE); ERC: Chronic Kidney Disease; EuroSCORE: European System for Cardiac Operative Risk Evaluation; FA: Atrial Fibrillation; FEVI: Left Ventricular Ejection Fraction; Grad.: Gradient; IC: Heart Failure; IM: Myocardial Infarction; NA: Not Applicable/Not Available; RVAT: Transcatheter Aortic Valve Replacement (TAVR); STS-PROM: Society of Thoracic Surgeons—Predicted Risk of Mortality; TFGe: Estimated Glomerular Filtration Rate; VARC: Valve Academic Research Consortium.

**Table 2:** Jadad scale for RCT’s.

Study	Randomization (0–2)	Blinding (0–2)	Withdrawals/Dropouts (0–1)	Total Score (de 5)
Reardon et al., 2017 [58]	2	0	1	3
Dangas et al., 2020 [35]	2	0	1	3
Leon et al., 2016 [51]	2	0	1	3
Thyregod et al., 2015 [67]	2	0	1	3
Mack et al., 2023 [54]	2	0	1	3
Brouwer et al., 2020 [30]	2	0	1	3
Adams et al., 2014 [18]	2	0	1	3
Blankenberg et al., 2024 [27]	2	0	1	3
Kapadia et al., 2014 [44]	2	0	1	3
Thyregod et al., 2024 [61]	2	0	1	3
Webb et al., 2015 [73]	2	0	1	3

**Table 3:** Assessment of risk of bias in observational studies using the ROBINS-E tool.

Study	Confounding	Participant Selection	Classification of Interventions	Deviations from Interventions	Missing Data	Measurement of Outcomes	Selection of Reported Outcomes	Overall Risk
Alasnag et al., 2022 [19]	Low	Low	Low	Low	Low	Low	Low	Low
Alnabti et al., 2025 [20]	Moderate	Low	Low	Low	Moderate	Low	Moderate	Moderate
Arnold et al., 2021 [21]	Low	Low	Low	Low	Moderate	Low	Low	Low
Azevedo et al., 2018 [22]	Moderate	Moderate	Low	Low	Moderate	Moderate	Moderate	Moderate
Barbanti et al., 2019 [23]	Moderate	Moderate	Low	Low	Low	Moderate	Low	Moderate
Bekeredjian et al., 2019 [24]	Moderate	Moderate	Low	Low	Low	Moderate	Low	Moderate
Beyersdorf et al., 2021 [25]	Moderate	Moderate	Low	Low	Low	Moderate	Moderate	Moderate
Biviano et al., 2017 [26]	Moderate	Low	Low	Moderate	Moderate	Low	Moderate	Moderate
Bouleti et al., 2015 [28]	Moderate	Low	Low	Low	Low	Moderate	Moderate	Moderate
Boute et al., 2025 [29]	Moderate	Low	Low	Low	Low	Low	Low	Moderate
Butt et al., 2021 [31]	Moderate	Low	Low	Low	Low	Low	Low	Moderate
Carroll et al., 2020 [32]	Low	Low	Low	Low	Low	Low	Low	Low
Chopard et al., 2015 [33]	Moderate	Low	Low	Moderate	Low	Moderate	Moderate	Moderate
Costa et al., 2024 [34]	Moderate	Low	Low	Low	Moderate	Moderate	Low	Moderate
Didier et al., 2021 [36]	Moderate	Low	Low	Low	Low	Moderate	Low	Moderate
Duncan et al., 2015 [37]	Moderate	Moderate	Low	Low	Low	Moderate	Moderate	Moderate
Frerker et al., 2017 [38]	Moderate	Moderate	Low	Low	Low	Moderate	Low	Moderate
Gilard et al., 2016 [39]	Moderate	Low	Low	Low	Low	Moderate	Low	Moderate
Goel et al., 2023 [40]	Moderate	Low	Low	Low	Low	Low	Low	Moderate
Holmes Jr. et al., 2015 [41]	Moderate	Low	Low	Low	Low	Low	Low	Moderate
Jochheim et al., 2019 [42]	Moderate	Low	Low	Low	Moderate	Moderate	Moderate	Moderate
Kaneko et al., 2022 [43]	Moderate	Low	Low	Low	Low	Low	Low	Moderate
Karra et al., 2024 [45]	Moderate	Low	Low	Low	Low	Low	Low	Moderate
Kawashima et al., 2020 [46]	Moderate	Low	Low	Low	Low	Low	Low	Moderate
Khashaba et al., 2014 [47]	Moderate	Low	Low	Low	Low	Low	Low	Moderate
Krasniqi et al., 2024 [48]	Moderate	Low	Low	Low	Low	Low	Low	Moderate
Lachonius et al., 2025 [49]	Moderate	Low	Low	Low	Low	Low	Low	Moderate
Lange et al., 2016 [50]	Moderate	Low	Low	Low	Low	Moderate	Moderate	Moderate
Li et al., 2021 [52]	Moderate	Low	Low	Low	Low	Low	Low	Moderate
Lundahl et al., 2024 [53]	Moderate	Low	Low	Low	Low	Low	Low	Moderate
Maeda et al., 2022 [55]	Moderate	Low	Low	Low	Low	Low	Low	Moderate
Mangner et al., 2019 [56]	Moderate	Low	Low	Low	Low	Low	Low	Moderate
Okuno et al., 2023 [57]	Moderate	Low	Low	Low	Low	Low	Low	Moderate
Reardon et al., 2017 [58]	Moderate	Low	Low	Low	Low	Low	Low	Moderate
Schaafsma et al., 2023 [59]	Moderate	Low	Low	Low	Low	Low	Low	Moderate
Seeger et al., 2017 [60]	Moderate	Low	Low	Low	Moderate	Moderate	Low	Moderate
Stortecky et al., 2019 [62]	Moderate	Low	Low	Moderate	Low	Moderate	Moderate	Moderate
Strange et al., 2022 [63]	Moderate	Low	Low	Low	Low	Low	Low	Moderate
Tamburino et al., 2015 [64]	Moderate	Moderate	Low	Low	Low	Moderate	Low	Moderate
Ternacle et al., 2021 [65]	Moderate	Low	Low	Low	Low	Low	Low	Moderate
Thogata et al., 2023 [66]	Moderate	Low	Low	Low	Low	Low	Low	Moderate
Tomii et al., 2025 [68]	Moderate	Low	Low	Low	Moderate	Moderate	Moderate	Moderate
Van Bergeijk et al., 2025 [69]	Moderate	Low	Low	Low	Low	Low	Low	Moderate
Vekstein et al., 2025 [70]	Moderate	Low	Low	Low	Low	Low	Low	Moderate
Virtanen et al., 2019 [71]	Moderate	Low	Low	Low	Low	Low	Low	Moderate
Wang et al., 2022 [72]	Moderate	Low	Low	Low	Low	Low	Low	Moderate
Witberg et al., 2019 [3]	Moderate	Low	Low	Low	Low	Low	Low	Moderate
Yu et al., 2018 [74]	Moderate	Low	Low	Low	Low	Low	Low	Moderate

**Table 4:** Assessment of methodological quality of observational studies using the Newcastle–Ottawa Scale (NOS).

Study	Selection (0–4★)	Comparability (0–2★)	Outcome (0–3★)	Total (0–9★)	Quality
Alasnag et al., 2022 [19]	★★★★	★★	★★★	9★	Excellent
Alnabti et al., 2025 [20]	★★★★	★★	★	7★	Good
Arnold et al. 2021 [21]	★★★★	★★	★★★	9★	Excellent
Azevedo et al., 2018 [22]	★★★	★	★★	6★	Good
Barbanti et al., 2019 [23]	★★★	★★	★★★	8★	Very Good
Bekeredjian et al., 2019 [24]	★★★	★★	★★★	8★	Very Good
Beyersdorf et al., 2021 [25]	★★★	★★	★★★	8★	Very Good
Biviano et al., 2017 [26]	★★★	★★	★★★	8★	Very Good
Bouleti et al., 2015 [28]	★★★	★	★★★	7★	Good
Boute et al., 2025 [29]	★★★★	★★	★★★	9★	Excellent
Butt et al., 2021 [31]	★★★	★★	★★★	8★	Very Good
Carroll et al., 2020 [32]	★★★★	★★	★★★	9★	Excellent
Chopard et al., 2015 [33]	★★★	★★	★★★	8★	Very Good
Costa et al., 2024 [34]	★★★	★★	★★	7★	Good
Didier et al., 2021 [36]	★★★★	★★	★★★	9★	Excellent
Duncan et al., 2015 [37]	★★★	★	★★★	7★	Good
Frerker et al., 2017 [38]	★★★	★★	★★★	8★	Very Good
Gilard et al., 2016 [39]	★★★★	★★	★★★	9★	Excellent
Goel et al., 2023 [40]	★★★★	★★	★★★	9★	Excellent
Holmes Jr. et al., 2015 [41]	★★★★	★★	★★★	9★	Excellent
Jochheim et al., 2019 [42]	★★★	★★	★★★	8★	Very Good
Kaneko et al., 2022 [43]	★★★★	★★	★★★	9★	Excellent
Karra et al., 2024 [45]	★★★★	★★	★★★	9★	Excellent
Kawashima et al., 2020 [46]	★★★★	★★	★★★	9★	Excellent
Khashaba et al., 2014 [47]	★★★★	★★	★★★	9★	Excellent
Krasniqi et al., 2024 [48]	★★★★	★★	★★★	9★	Excellent
Lachonius et al., 2025 [49]	★★★★	★★	★★★	9★	Excellent
Lange et al., 2016 [50]	★★★	★★	★★★	8★	Very Good
Li et al., 2021 [52]	★★★★	★★	★★★	9★	Excellent
Lundahl et al., 2024 [53]	★★★★	★★	★★★	9★	Excellent
Maeda et al., 2022 [55]	★★★★	★★	★★★	9★	Excellent
Mangner et al., 2019 [56]	★★★	★★	★★★	8★	Very Good
Okuno et al., 2023 [57]	★★★★	★★	★★★	9★	Excellent
Reardon et al., 2017 [58]	★★★★	★★	★★★	9★	Excellent
Schaafsma et al., 2023 [59]	★★★★	★★	★★★	9★	Excellent
Seeger et al., 2017 [60]	★★★	★★	★★★	8★	Very Good
Stortecky et al., 2019 [62]	★★★	★	★★★	7★	Good
Strange et al., 2022 [63]	★★★★	★★	★★★	9★	Excellent
Tamburino et al., 2015 [64]	★★★	★★	★★★	8★	Very Good
Ternacle et al., 2021 [65]	★★★★	★★	★★★	9★	Excellent
Thogata et al., 2023 [66]	★★★★	★★	★★★	9★	Excellent
Tomii et al., 2025 [68]	★★★	★★	★★★	8★	Very Good
Van Bergeijk et al., 2025 [69]	★★★★	★★	★★★	9★	Excellent
Vekstein et al., 2025 [70]	★★★★	★★	★★★	9★	Excellent
Virtanen et al., 2019 [71]	★★★★	★★	★★★	9★	Excellent
Wang et al., 2022 [72]	★★★★	★★	★★★	9★	Excellent
Witberg et al., 2019 [3]	★★★★	★★	★★★	9★	Excellent
Yu et al., 2018 [74]	★★★★	★★	★★★	9★	Excellent

**Table 5:** Study-level characteristics database used for meta-learning analysis.

Study ID	1-Year Mortality Rate (%)	Average Age	% Women	% Diabetes	% CKD	% AF	Average STS-PROM	Average EuroSCORE II	Average LVEF (%)	% Transfemoral	% Self-Expanding	Average Recruitment Year	Cohort Size	Study Design	Country	Quality Score
Adams et al., 2014 [18]	14.2	83.1	46.9	34.9	12.2	40.9	7.3	17.7	NA	82.8	100.0	2013	390	RCT	USA	6.0
Alasnag et al., 2022 [19]	5.4	74.6	44.0	61.6	8.4	18.1	4.9	NA	53.0	95.8	61.8	2018	795	Registry	Gulf Region	10.0
Alnabti et al., 2025 [20]	8.7	73.7	47.7	76.3	30.3	27.4	NA	NA	NA	99.2	77.2	2018	241	Registry	Qatar	7.8
Arnold et al., 2020 [21]	12.4	83.0	46.7	36.5	NA	40.6	5.8	NA	58.0	91.5	NA	2016	45,884	Registry	USA	10.0
Azevedo et al., 2018 [22]	17.2	77.8	62.1	24.4	72.4	22.4	NA	12.7	57.4	81.0	68.9	2013	58	Observational	Brazil	6.7
Barbanti et al., 2019 [23]	13.8	80.5	58.9	24.8	1.4	NA	NA	4.9	53.6	100.0	55.1	2011	650	Observational (PSM)	Italy	8.9
Bekeredjian et al., 2019 [24]	10.0	78.9	39.1	21.4	0.0	18.8	2.9	12.9	54.6	83.1	NA	2015	6062	Registry	Germany	8.9
Beyersdorf et al., 2021 [25]	20.9	80.9	53.2	34.4	5.3	29.1	6.3	NA	NA	72.9	NA	2012	4157	Registry	Germany	8.9
Biviano et al., 2017 [26]	20.6	85.5	50.7	37.7	16.5	25.0	10.9	NA	56.9	58.4	0.0	2010	1879	Post-hoc of RCT	Multinational	8.9
Blankenberg et al., 2024 [27]	2.6	74.0	44.0	33.8	NA	28.9	1.8	2.1	57.8	97.3	35.1	2019	701	RCT	Germany	6.0
Bouleti et al., 2015 [28]	16.0	82.0	43.9	23.6	4.1	41.5	7.1	7.8	50.1	68.3	NA	2008	123	Observational	France	7.8
Boute et al., 2025 [29]	11.9	85.4	55.1	18.1	59.7	30.8	NA	4.3	59.8	92.2	80.7	2017	449	Observational	Belgium	10.0
Brouwer et al., 2020 [30]	6.0	80.0	48.7	24.5	NA	0.0	2.5	NA	NA	NA	NA	2016	665	RCT	Europe	6.0
Butt et al., 2021 [31]	11.0	82.5	46.3	22.3	11.7	100.0	NA	NA	NA	77.3	NA	2014	735	Registry	Denmark	8.9
Carroll et al., 2020 [32]	13.2	80.0	44.2	NA	NA	NA	4.4	NA	NA	95.3	26.7	2019	276,316	Registry	USA	10.0
Chopard et al., 2015 [33]	19.2	82.8	49.4	25.4	8.6	25.8	14.1	21.8	NA	73.0	33.5	2011	3875	Registry	France	8.9
Costa et al., 2024 [34]	10.1	82.0	56.4	28.3	9.7	25.0	3.2	NA	60.0	100.0	50.0	2019	1174	Registry	Multinational	7.8
Dangas et al., 2020 [35]	5.3	80.6	50.5	28.7	NA	0.0	4.2	4.1	57.8	NA	51.0	2017	1644	RCT	Multinational	6.0
Didier et al., 2021 [36]	14.9	83.2	48.9	26.2	34.7	100.0	5.0	NA	55.0	89.2	NA	2015	1714	Registry	France	10.0
Duncan et al., 2015 [37]	21.4	82.0	48.0	22.9	6.5	23.9	NA	18.5	NA	68.4	52.5	2008	850	Registry	United Kingdom	7.8
Frerker et al., 2020 [38]	7.4	77.5	60.4	23.6	10.6	8.0	NA	6.8	NA	100.0	NA	2014	805	Observational (PSM)	Germany	8.9
Gilard et al., 2016 [39]	23.2	82.8	49.3	25.4	2.5	26.3	NA	21.7	55.0	73.0	33.7	2011	4201	Registry	France	10.0
Goel et al., 2023 [40]	51.0	77.0	41.2	44.9	53.9	47.9	6.9	NA	25.0	88.5	35.6	2018	1172	Registry	USA	10.0
Holmes et al., 2015 [41]	23.7	84.0	49.3	42.1	8.8	38.6	11.2	NA	54.0	62.1	NA	2012	42,982	Registry	USA	10.0
Jochheim et al., 2019 [42]	13.8	81.0	60.7	30.1	40.9	100.0	4.2	NA	55.0	95.9	NA	2012	962	Registry	Europe	8.9
Kaneko et al., 2022 [43]	12.1	81.6	47.4	42.6	55.4	49.3	5.0	NA	NA	94.7	35.8	2015	64,815	Registry	USA	10.0
Kapadia et al., 2015 [44]	30.7	83.1	43.6	44.7	45.3	33.0	11.8	29.3	58.0	100.0	0.0	2008	179	RCT	Multinational	6.0
Karra et al., 2024 [45]	9.3	81.0	44.0	37.0	62.0	40.0	2.9	2.6	55.0	94.6	49.3	2014	2073	Registry	Israel	10.0
Kawashima et al., 2020 [46]	12.0	85.0	71.0	24.0	47.0	32.0	5.4	NA	62.0	96.6	62.4	2014	7981	Registry	Japan	10.0
Khashaba et al., 2014 [47]	20.0	78.6	60.0	40.0	60.0	50.0	NA	26.5	51.0	100.0	0.0	2012	10	Observational	Egypt	10.0
Krasniqi et al., 2024 [48]	6.6	82.0	51.0	23.0	36.0	38.0	NA	3.1	NA	100.0	58.0	2019	1146	Registry	Denmark	10.0
Lachonious et al., 2023 [49]	12.0	81.2	48.0	28.0	40.2	47.9	NA	3.8	NA	91.1	62.4	2012	3349	Registry	Sweden	10.0
Lange et al., 2016 [50]	20.7	81.1	58.7	32.1	35.8	36.1	7.5	23.3	NA	100.0	NA	2012	6563	Registry	Germany	8.9
Leon et al., 2016 [51]	12.3	81.6	44.9	31.6	64.1	37.0	5.8	NA	NA	76.3	0.0	2012	1011	RCT	Multinational	6.0
Li et al., 2021 [52]	6.8	74.8	47.9	29.8	29.8	13.9	4.1	NA	62.1	83.9	83.3	2016	3028	Registry	China	10.0
Lundahl et al., 2024 [53]	11.0	82.0	47.5	23.9	44.0	48.0	NA	2.9	NA	96.6	NA	2019	4847	Registry	Denmark	10.0
Mack et al., 2023 [54]	1.0	73.0	32.5	30.1	NA	15.3	1.9	NA	60.9	100.0	0.0	2017	496	RCT	Multinational	6.0
Maeda et al., 2022 [55]	9.7	85.3	68.3	23.3	47.9	32.1	4.6	NA	62.0	96.6	NA	2015	16,837	Registry	Japan	10.0
Mangner et al., 2019 [56]	12.6	81.5	58.3	32.6	47.6	100.0	4.2	NA	52.5	100.0	NA	2014	598	Observational	Germany	8.9
Okuno et al., 2023 [57]	6.7	82.2	41.5	23.9	43.1	40.5	3.1	NA	58.0	95.8	45.4	2016	11,889	Registry	Switzerland	10.0
Reardon et al., 2017 [58]	6.7	79.8	44.1	32.8	32.2	22.8	4.4	NA	NA	90.7	100.0	2014	864	RCT	Multinational	6.0
Schaafsma et al., 2023 [59]	8.1	73.1	42.4	NA	19.0	19.3	5.7	5.2	NA	95.0	NA	2017	2083	Registry	South Africa	10.0
Seeger et al., 2017 [60]	13.9	82.5	61.8	32.7	64.0	100.0	4.7	21.0	54.0	100.0	63.2	2013	272	Observational	Germany	8.9
Thyregod et al., 2024 [61]	4.9	79.1	48.2	15.6	9.9	7.1	3.0	7.5	60.1	100.0	100.0	2011	145	RCT	Nordic Countries	6.0
Stortecky et al., 2019 [62]	16.7	83.0	46.1	23.9	20.4	22.8	7.5	22.8	55.0	71.8	92.5	2011	697	Registry	Switzerland	7.8
Strange et al., 2022 [63]	8.1	82.0	48.6	22.8	43.1	47.9	NA	2.9	NA	96.0	62.1	2014	6,56	Registry	Denmark	10.0
Tamburino et al., 2015 [64]	13.8	80.5	58.9	24.8	1.4	NA	NA	21.4	53.6	100.0	55.1	2011	650	Observational (PSM)	Italy	8.9
Ternacle et al., 2021 [65]	1.0	73.3	32.6	30.1	NA	15.3	1.9	NA	60.8	100.0	0.0	2017	495	Sub-study of RCT	Multinational	10.0
Thogata et al., 2023 [66]	18.2	72.8	41.5	44.8	24.3	22.4	NA	NA	43.1	78.4	62.7	2019	302	Observational	India	10.0
Thyregod et al., 2015 [67]	4.9	79.1	48.2	15.6	9.9	7.1	3.0	NA	60.1	100.0	100.0	2011	145	RCT	Nordic Countries	6.0
Tomii et al., 2025 [68]	7.7	80.0	60.5	23.3	35.3	14.1	2.9	NA	58.0	100.0	50.0	2017	739	Sub-study of RCT	Europe	8.9
Van Bergeijk et al., 2025 [69]	5.6	81.0	48.3	21.1	33.6	29.8	NA	2.6	NA	95.8	NA	2017	10,767	Registry	Netherlands	10.0
Vekstein et al., 2025 [70]	5.7	75.0	37.5	32.2	NA	22.8	2.0	NA	58.0	100.0	32.2	2022	16,988	Registry	USA	10.0
Virtanen et al., 2019 [71]	4.1	75.3	40.5	20.3	6.3	19.0	1.8	NA	NA	100.0	NA	2015	306	Observational (PSM)	Finland	10.0
Wang et al., 2022 [72]	6.7	76.8	51.7	30.0	NA	14.2	6.1	NA	57.9	99.2	100.0	2020	120	Prospective	China	10.0
Webb et al., 2015 [73]	22.0	84.1	40.2	42.1	49.6	38.6	10.8	NA	54.0	100.0	0.0	2011	271	RCT	Multinational	6.0
Witberg et al., 2019 [3]	5.1	65.0	30.4	38.6	NA	10.9	2.3	1.9	50.0	96.6	62.1	2018	750	Registry	Multinational	10.0
Yu et al., 2018 [74]	10.6	80.0	49.3	35.5	23.3	19.3	5.8	17.5	58.6	91.6	NA	2014	1007	Registry	South Korea	10.0

Abbreviations—ERC: Chronic Kidney Disease; FA: Atrial Fibrillation; STS-PROM: Society of Thoracic Surgeons Predicted Risk of Mortality; FEVI: Left Ventricular Ejection Fraction; RCT: Randomized Controlled Trial; PSM: Propensity Score Matching; NA: Not Available.

**Table 6:** Performance metrics of different regression models.

Model Abbr.	Model Name	MAE	MSE	RMSE	R^2^	RMSLE	MAPE	TT (s)
ada	AdaBoost Regressor	41.837	588.412	58.768	0.1908	0.4504	0.3665	0.1230
xgboost	Extreme Gradient Boosting	44.553	690.457	63.180	0.0308	0.4699	0.3390	0.1010
et	Extra Trees Regressor	46.728	774.489	65.289	−0.0353	0.4805	0.3757	0.1570
rf	Random Forest Regressor	46.907	693.559	64.722	−0.0126	0.4933	0.4015	0.2430
gbr	Gradient Boosting Regressor	47.496	801.806	67.985	−0.1607	0.5074	0.3751	0.1080
br	Bayesian Ridge	52.424	728.165	70.859	−0.5175	0.5354	0.7187	0.0640
lasso	Lasso Regression	53.373	759.845	71.738	−0.5479	0.5573	0.7428	0.0590
llar	Lasso Least Angle Regression	53.373	759.845	71.738	−0.5479	0.5573	0.7428	0.0590
huber	Huber Regressor	54.631	858.332	75.655	−1.1585	0.5549	0.6779	0.0960
en	Elastic Net	55.280	811.797	75.077	−0.7140	0.5632	0.6957	0.0630
dt	Decision Tree Regressor	55.640	783.749	74.446	−0.4875	0.5958	0.4110	0.0850
ridge	Ridge Regression	58.226	1,123.217	78.645	−1.0956	0.6054	0.8556	0.0600
lr	Linear Regression	60.527	959.125	84.368	−1.8527	0.6060	0.8607	0.0610
lightgbm	Light Gradient Boosting Machine	60.914	829.995	80.008	−0.8215	0.6483	0.7869	0.1820
dummy	Dummy Regressor	60.914	929.995	81.239	−0.8433	0.6483	0.7869	0.0000
omp	Orthogonal Matching Pursuit	65.273	950.847	82.094	−1.0051	0.6641	0.8143	0.0570
knn	K Neighbors Regressor	67.586	963.289	84.364	−1.4425	0.7194	0.8750	0.0590
lar	Least Angle Regression	151.363	13,529.595	231.197	−38.9906	0.8156	15.673	0.0660
par	Passive Aggressive Regressor	1,591.696	7,776,826.429	3,068.901	−40,213.4769	13.979	127.703	0.0610

Abbreviations—ada: AdaBoost Regressor; xgboost: Extreme Gradient Boosting; et: Extra Trees Regressor; rf: Random Forest Regressor; gbr: Gradient Boosting Regressor; br: Bayesian Ridge; lasso: Lasso Regression; llar: Lasso Least Angle Regression; huber: Huber Regressor; en: Elastic Net; dt: Decision Tree Regressor; ridge: Ridge Regression; lr: Linear Regression; lightgbm: Light Gradient Boosting Machine; dummy: Dummy Regressor; omp: Orthogonal Matching Pursuit; knn: K Neighbors Regressor; lar: Least Angle Regression; par: Passive Aggressive Regressor.

## Data Availability

The original contributions presented in this study are included in the article. Further inquiries can be directed to the corresponding author(s).

## Abbreviations

The following abbreviations are used in this manuscript:

TAVR	Transcatheter Aortic Valve Replacement
STS-PROM	Society of Thoracic Surgeons Predicted Risk of Mortality
LVEF	Left Ventricular Ejection Fraction
MACE	Major Adverse Cardiac Events
RCT	Randomized Controlled Trial
NOS	Newcastle–Ottawa Scale
ROBINS-E	Risk of Bias in Non-randomised Studies—of Exposures
RR	Risk Ratio
MAE	Mean Absolute Error
RMSE	Root Mean Squared Error
AI	Artificial Intelligence
IPD	Individual Patient Data
VARC	Valve Academic Research Consortium
AF	Atrial Fibrillation
CKD	Chronic Kidney Disease
CAD	Coronary Artery Disease
